# Efficient solid-state photoswitching of methoxyazobenzene in a metal–organic framework for thermal energy storage†

**DOI:** 10.1039/d2sc00632d

**Published:** 2022-02-23

**Authors:** Kieran Griffiths, Nathan R. Halcovitch, John M. Griffin

**Affiliations:** Department of Chemistry, Lancaster University Lancaster LA14YB UK j.griffin@lancaster.ac.uk; Materials Science Institute, Lancaster University Lancaster LA14YB UK

## Abstract

Efficient photoswitching in the solid-state remains rare, yet is highly desirable for the design of functional solid materials. In particular, for molecular solar thermal energy storage materials high conversion to the metastable isomer is crucial to achieve high energy density. Herein, we report that 4-methoxyazobenzene (MOAB) can be occluded into the pores of a metal–organic framework Zn_2_(BDC)_2_(DABCO), where BDC = 1,4-benzenedicarboxylate and DABCO = 1,4-diazabicyclo[2.2.2]octane. The occluded MOAB guest molecules show near-quantitative *E* → *Z* photoisomerization under irradiation with 365 nm light. The energy stored within the metastable *Z*-MOAB molecules can be retrieved as heat during thermally-driven relaxation to the ground-state *E*-isomer. The energy density of the composite is 101 J g^−1^ and the half-life of the *Z*-isomer is 6 days when stored in the dark at ambient temperature.

## Abstract 2

4-Methoxyazobenzene can be occluded into the pores of a MOF and show near-quantitative *E* → *Z* photoisomerization under irradiation with 365 nm light. The energy density of the composite is 101 J g^−1^ and the half-life of the *Z*-isomer is 6 days.

## Graphical abstract

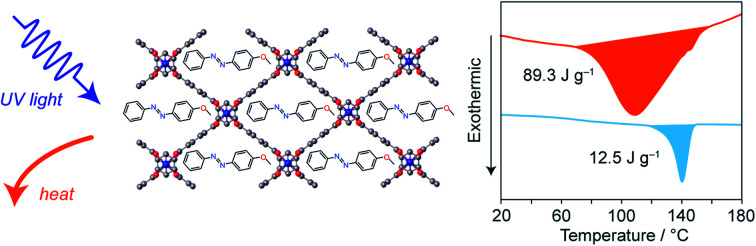

## Introduction

Molecular photoswitches are currently receiving significant interest for molecular solar thermal (MOST) energy storage applications. MOST materials convert photon energy to thermal energy through reversible isomerization between ground and metastable isomeric states.^1–3^ While many classes of photoswitch have been investigated for MOST applications, azobenzene (AB) derivatives are among the most widely studied^4^ due to their high quantum yield, high-fatigue resistance and appreciable energy separation between the ground-state *E* and metastable *Z* isomers. However, in their pure solid form, photoswitching of AB derivatives is often limited due to dense crystal packing. Several strategies have been proposed to address this problem by increasing the molecular free volume; these include templating AB on nanotubes^5–7^ and graphene,^8,9^ incorporating as sidegroups within polymers,^10–12^ and using bulky functional groups to form amorphous films.^10^ Recently, confinement within metal–organic frameworks (MOFs) has been shown as an effective way to impart conformational freedom to photoswitches within bulk solid materials. Using this approach, solid-state photoswitching has been demonstrated for AB derivatives^13–22^ as well as other molecules including dithienylethenes,^23–27^ 2-phenylazopyridine,^28^ and spiropyrans.^29–35^

In addition to spatial considerations, another requirement for solid-state MOST materials is to ensure a high degree of photoconversion to the metastable state. For AB, overlap of the π–π* absorption bands for the *E* and *Z* isomers means that the photostationary state (PSS) is limited to approximately 78% *Z* isomer under 365 nm irradiation.^36^ The effects of MOF confinement on the achievable PSS are not well understood but recent studies have shown it can be detrimental depending on the structural properties of the MOF.^20,37^ One way to increase the intrinsic PSS is to alter the electronic structure through chemical modification. This has been demonstrated for *ortho*-functionalised AB derivatives, for which the *E* and *Z* isomers have well-separated n–π* bands.^38^ However, while this can lead to more efficient photoswitching, including quantitative switching within MOFs,^39^ it can also reduce the energy difference between the ground and metastable states, thereby reducing the energy density of the resulting MOST material.^1,37,39,40^ The design of both the photoswitch and MOF architecture therefore need to be carefully considered in order to achieve a high degree of conversion as well as a high energy density.

Herein, we report the structural and photothermal properties of a composite comprising the breathable MOF Zn_2_(BDC)_2_(DABCO) (1) and 4-methoxyazobenzene (MOAB) which shows promising properties for solid-state MOST applications. This composite (1⊃MOAB), has the advantages that 1 is straightforwardly prepared by solvothermal synthesis, and MOAB is widely commercially available. Unlike bulk MOAB which photoconverts to 70% *Z*-isomer, MOAB exhibits a photostationary state of nearly 100% when confined within the composite, resulting in an energy density of 101 J g^−1^. The half-life of the *Z*-isomer within the composite is 6 days when stored in the dark at ambient temperature.

## Results and discussion

**Fig. 1:**
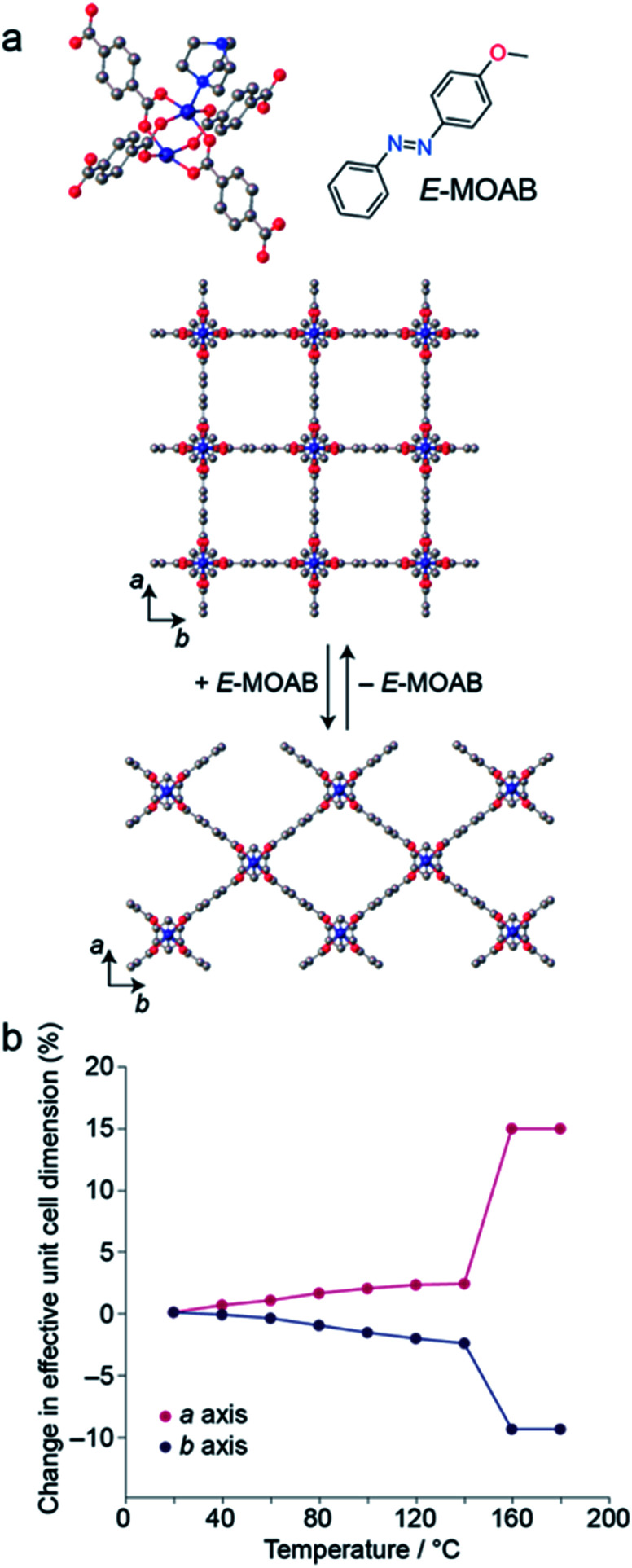
(a) Schematic representation of Zn_2_(BDC)_2_(DABCO) (1), showing the transition between the tetragonal and orthorhombic structures upon uptake of MOAB. (b) Change in *a* and *b* unit cell dimensions of 1⊃MOAB as a function of temperature.

MOAB was occluded within the pores of 1 by a previously described melt-infiltration procedure.^13,37^ The maximum loading level obtained was 1.25 MOAB molecules per pore (Fig. S1, S2 and Tables S1–S3†). DSC measurements confirm the absence of residual MOAB outside the pores (Fig. S3†). Surprisingly, this loading level is comparable to the 1.3 molecules per pore observed for AB within 1 (1⊃AB), despite the increased length of the guest molecule.^37^1 is known to undergo guest-induced breathing,^41^ and X-ray powder diffraction (XRPD) confirmed that the framework within 1⊃MOAB is contracted compared to the guest-free form (Fig. 1a). Profile fitting shows a single phase corresponding to the narrow pore (np) orthorhombic (*Cmmm*) structure (Table 1, Fig. S4 and S5†). This symmetry contrasts with the tetragonal phase previously reported^13^ for 1⊃AB when one molecule of AB is occluded per pore, though it is less contracted, presumably due to the high density of occluded guest molecules. This is notable because the cell parameters of this orthorhombic phase are highly sensitive to changes in temperature (Fig. 1b), which indicates flexibility in the low temperature phase of the framework. Additionally, variable-temperature (VT-) XRPD shows that 1⊃MOAB undergoes a np to large pore (lp) phase transition at 160 °C, where the lp structure is equivalent to the guest-free phase (Fig. 1b). Table 1 summarizes the lattice parameters for the different phases of 1⊃MOAB studied.

**Table 1:** Space groups and unit cell volumes for the observed phases of 1⊃MOAB. np = narrow pore; lp = large pore; irr = irradiated

	Space group	Unit cell volume (Å^3^)	Δ*V* (%)
1⊃MOAB (np)	*Cmmm*	2236.8^a^	—
1⊃MOAB (lp)	*P*4/*mmm*	1170.4	4.3
1⊃MOAB (irr)	*P*4/*mmm*	1159.0	3.5

a For *Cmmm*, *Z* = 4; whereas for *P*4/*mmm*, *Z* = 2.

VT-XRPD experiments below 140 °C show a reversible temperature-dependent expansion and contraction of the *a* and *b* cell lengths, respectively (Fig. 1b, Tables S4 and S5†); however, the *c* cell length (defined by the DABCO pillar group) remains consistent across the temperature range (Fig. S6–S13†). For an alkoxy-functionalised analogue of 1, Henke *et al.* reported expansion of the *a* and *b* cell lengths with increasing temperature below the np → lp phase transition, with a concomitant increase in the unit cell volume.^42^ However, for 1⊃MOAB we observe a small volume contraction between 80 and 140 °C, which is driven by the contraction of the *b* axis. This highlights that the arrangement of guest-molecules can significantly affect the flexibility of 1.

**Fig. 2:**
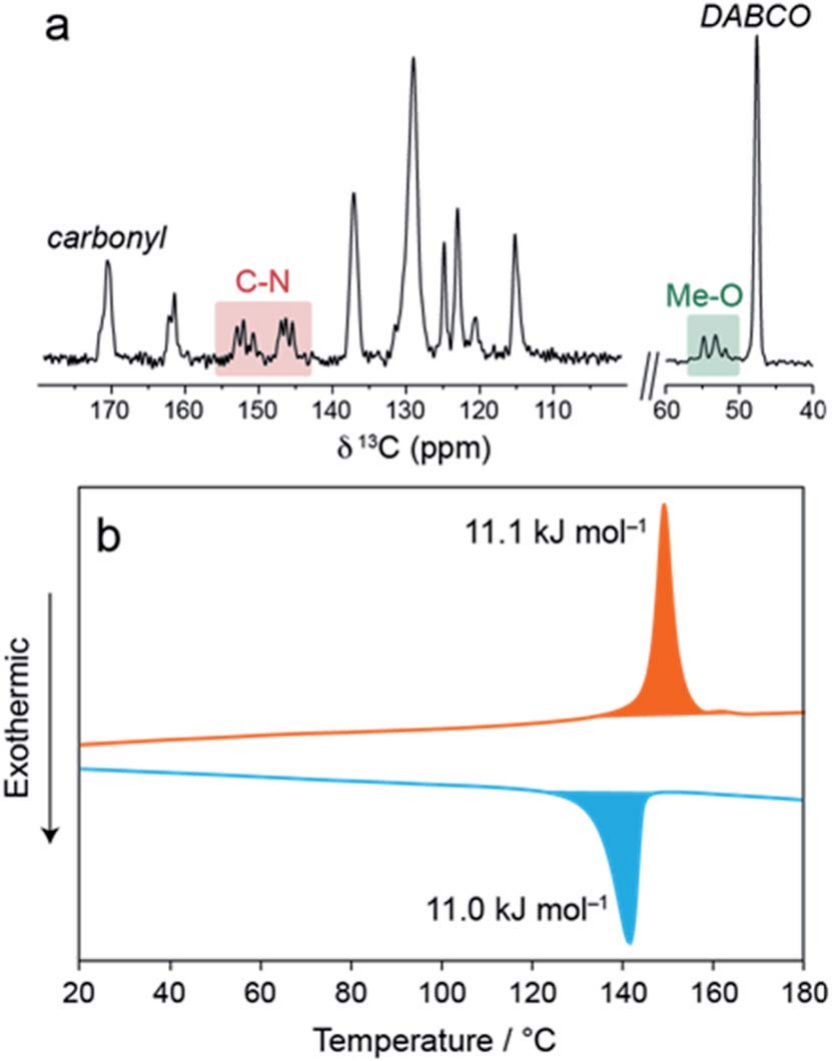
(a) ^13^C CPMAS NMR spectrum of 1⊃MOAB. (b) DSC thermogram of 1⊃MOAB showing the first heat (red) and the first cool (blue).

In the ^13^C cross-polarisation (CP) magic-angle spinning (MAS) NMR spectrum of 1⊃MOAB (Fig. 2a and S14–S16†), single DABCO and carbonyl resonances at 47.6 and 171.2 ppm are consistent with those previously observed for the np orthorhombic structure of 1⊃AB.^13^ Considering the MOAB guest molecules, the methoxy and C–N carbons each show three distinct resonances, suggesting three crystallographically inequivalent MOAB molecular conformations within the pores. Comparison with DFT chemical shift calculations (Tables S6 and S7†) suggests fast-rotational dynamics around the molecular axis, as was previously observed for 1⊃AB.^13^ A spectrum recorded at −34 °C showed a pronounced broadening of the MOAB ring resonances consistent with a reduction in the timescale of this motion (Fig. S17 and S18†). The dynamics of the guest molecules indicates that despite the dense packing, a significant degree of conformational freedom is retained.

Thermal analysis of 1⊃MOAB between 0–200 °C (Fig. 2b, Fig. S19, Tables S8 and S9†) shows a reversible phase transition consistent with the np → lp phase transition observed by XRPD. The onset temperature is 141 °C and the enthalpy is 11.1 kJ mol^−1^. Both of these values are lower than observed for 1⊃AB (21.4 kJ mol^−1^ and 160 °C), implying weaker host–guest interactions in 1⊃MOAB.

**Fig. 3:**
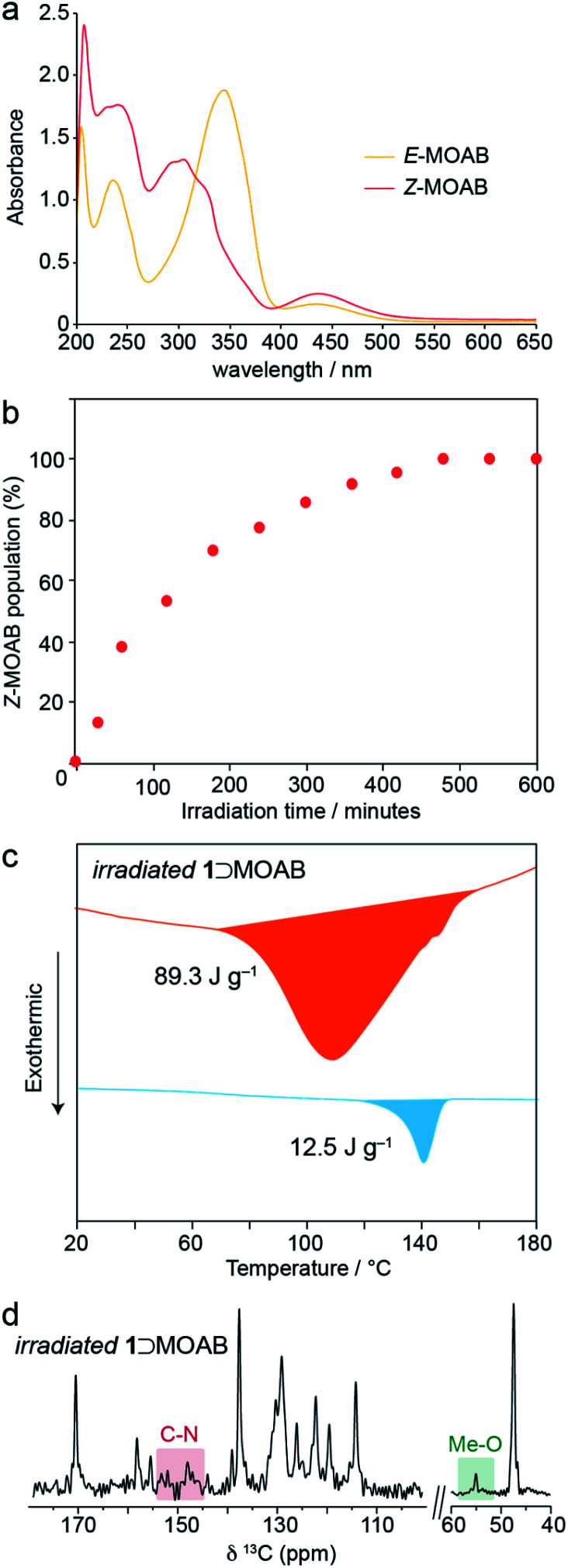
(a) UV-vis spectra of *E*-MOAB and *Z*-MOAB recorded in benzene solution. (b) *Z*-MOAB population as a function of UV irradiation time for 1⊃MOAB as measured by ^1^H NMR. (c) DSC thermogram of irradiated 1⊃MOAB showing the first heat (red) and the first cool (blue). (d) ^13^C CPMAS NMR spectrum of irradiated 1⊃MOAB.

The solution-state UV-vis spectrum of *E*-MOAB (Fig. 3a) shows characteristic absorptions at 348 nm (π–π*) and 440 nm (n–π*). The π–π* absorption is significantly red-shifted relative to *E*-AB (320 nm); this is attributed to the electron donating effect of the methoxy group in MOAB. Irradiation with 365 nm light causes *E* → *Z* isomerization, resulting in a decrease and shift of the π–π* absorption to 305 nm and producing a photostationary state containing 98% *Z*-MOAB, as measured by ^1^H NMR. The near-quantitative photoswitching can be attributed to the significant redshift of the π–π* absorption in *E*-MOAB which effectively separates the absorptions for the *E* and *Z* isomers.

Irradiation of 1⊃MOAB at 365 nm causes *E* → *Z* isomerization within the framework and a ^1^H NMR shows that a photostationary state (PSS) of 98% is reached after 480 minutes (Fig. 3b). Crystalline MOAB photomelts under 365 nm light;^43^ however, 1⊃MOAB remained solid and thermal measurements show MOAB is not lost from the pores. The PSS of the composite is equivalent to the solution-state value (Fig. S20–S25†) and higher than the ∼70% PSS obtained when photo-melting pure MOAB (Fig. S22†). It is noteworthy that the high PSS of MOAB is maintained when confined within 1, whereas other guest molecules show a significantly reduced PSS (AB – 40%, PAP – 28%).^13,22,28^ However, recent work has shown that fluorinated ABs can also be quantitatively switched inside MOFs.^21^ This highlights that further investigation is required to understand the precise factors controlling the PSS which may include the density and arrangement of the guest molecules as well as guest-induced breathing of the framework.

**Fig. 4:**
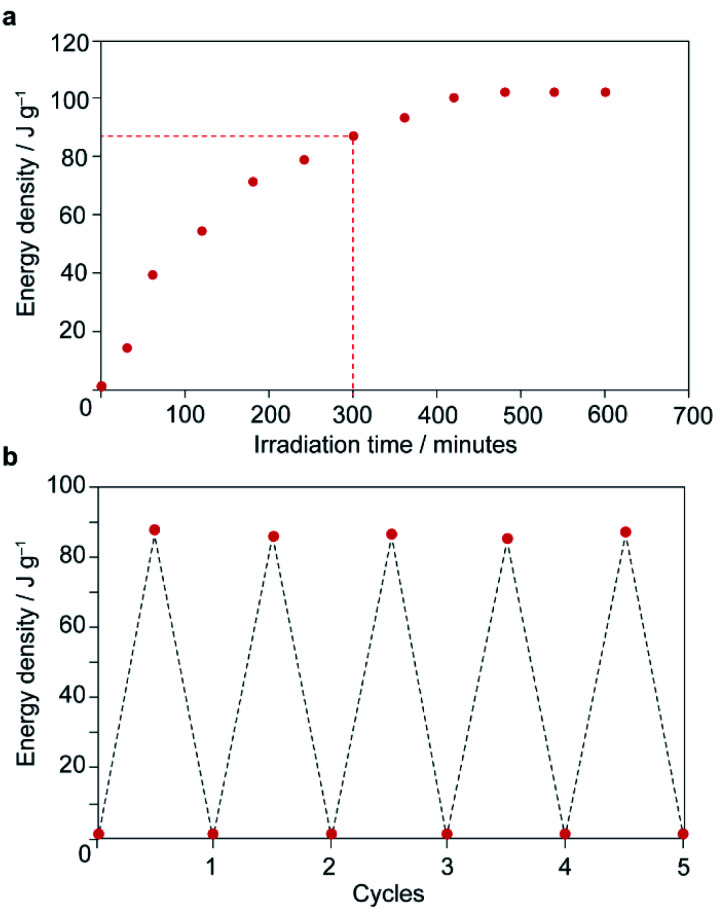
(a) Net energy storage of irradiated 1⊃MOAB as a function of irradiation time. Dotted line shows irradiation time used for (b). (b) Net energy storage of irradiated 1⊃MOAB over multiple irradiation-discharge cycles.

Thermal analysis of the irradiated composite between 0–200 °C reveals a large exotherm upon the first heating branch (Fig. 3c), which is attributed to thermally-driven reconversion of the *Z*-MOAB molecules to the ground-state *E* isomer. The exotherm magnitude agrees well with the calculated the *Z* → *E* reconversion enthalpy based on the PSS and a DFT-calculated *E*–*Z* energy difference of 68.7 kJ mol^−1^ (Fig. S26–S28 and Tables S10–S13†).^43^ On the cooling branch, another exothermic feature is observed corresponding to the lp → np phase transition (Fig. S29†). This feature is expected due to contraction of the lp framework around the *E*-MOAB formed due to *Z* → *E* reconversion that has taken place on the heating branch. Over one full heating and cooling cycle, the exotherms for *Z* → *E* reconversion and the lp → np phase transition give a combined energy density of 86.4 kJ mol^−1^ or 101 J g^−1^. The cyclability of the composite was investigated using a reduced irradiation time of 300 minutes which results in a conversion to 83% *Z*-MOAB, with a corresponding energy density of 86.4 J g^−1^ (Fig. 4a). This energy density was maintained over five full cycles of irradiation and thermally-driven discharge with no degradation of the composite or loss of MOAB from the pores of 1 (Fig. 4b).

The gravimetric energy density of 101 J g^−1^ for 1⊃MOAB represents an increase by a factor greater than 3 in comparison to the previously reported energy density of 28.9 J g^−1^ for 1⊃AB.^13^ This is due to a combination of the larger energy *E*–*Z* difference (68.7 kJ mol^−1^ for MOAB *vs.* ∼50 kJ mol^−1^ for AB) and the almost quantitative conversion to the *Z*-isomer of MOAB within the composite. The energy density of *Z*-MOAB is also comparable to other azobenzene-based solid-state MOST materials including functionalised polymers with reported energy densities between 90 and 176 J g^−1^,^10,11^ and an amorphous film formed from a bulky azobenzene derivative which showed an energy density of 135 J g^−1^.^12^ Complementing work on azopolymers and molecular AB derivatives, surface-templated azobenzene derivatives have been demonstrated to store up to a remarkable 540 J g^−1^, and in some cases also report half-lives of nearly 2 months.^9^ However, reports of energy densities around 300 J g^−1^ are more typical, with associated half-lives ranging from hours to days.^44^

One of the advantageous properties of 1⊃MOAB is near-quantitative isomerisation is achieved in the bulk material without the requirement to suspend in solution or cast into films or surface-based architectures. While near quantitative conversion within the bulk of a MOF has been previously reported for an *ortho*-fluoroazobenzene derivative confined within 1; however, DFT calculations (Table S14†) show that the functionalisation of the azobenzene moiety in this case is predicted to result in a significant decrease in the *E*–*Z* energy difference and therefore a marked reduction in energy density as compared to 1⊃MOAB.

To rationalize the high PSS within the composite, XRPD measurements were performed to monitor structural changes during UV irradiation. With increasing irradiation time, the reflections of the np orthorhombic phase shift and decrease in intensity and new reflections emerge (Fig. S30†). After 240 minutes the new phase dominates the pattern. Profile fitting shows this phase is consistent with the lp tetragonal (*P*4/*mmm*) structure (Table 1 and Fig. S31†). This is consistent with a previously reported phase change from orthorhombic np to tetragonal lp for irradiated of 1⊃AB.^22^ For 1⊃MOAB, the expansion of the framework by irradiation (3.5%) is lower than that of the temperature-induced np → lp phase transition (4.4%). We note that a minor component of the orthorhombic np structure is retained even after the PSS is reached. This suggests that a small proportion of the occluded MOAB molecules isomerize within pores that remain contracted. However, 1⊃MOAB shows greater overall flexibility than 1⊃AB, which is highlighted by the temperature-dependent distortion of the framework below the phase transition as well as the larger expansion upon irradiation. The differences in the guest-induced flexibility are presumably related to the ordering of the guest molecules and/or host–guest interactions, which may be key to achieving efficient photoconversion to the *Z* isomer.

A ^13^C CPMAS NMR spectrum of irradiated 1⊃MOAB (Fig. 3d, S32 and S33†) shows no changes in the chemical shifts of the framework resonances, although the carbonyl resonance sharpens. Considering the MOAB resonances, there is considerable shifting for each carbon site. The most noticeable of these is the methoxy carbon which reduces to a single resonance at 55.1 ppm. Comparison of the experimental chemical shifts with DFT-calculated values (Tables S15 and S16†) suggests that *Z*-MOAB molecules also undergo rapid rotational motion within the pores.

**Fig. 5:**
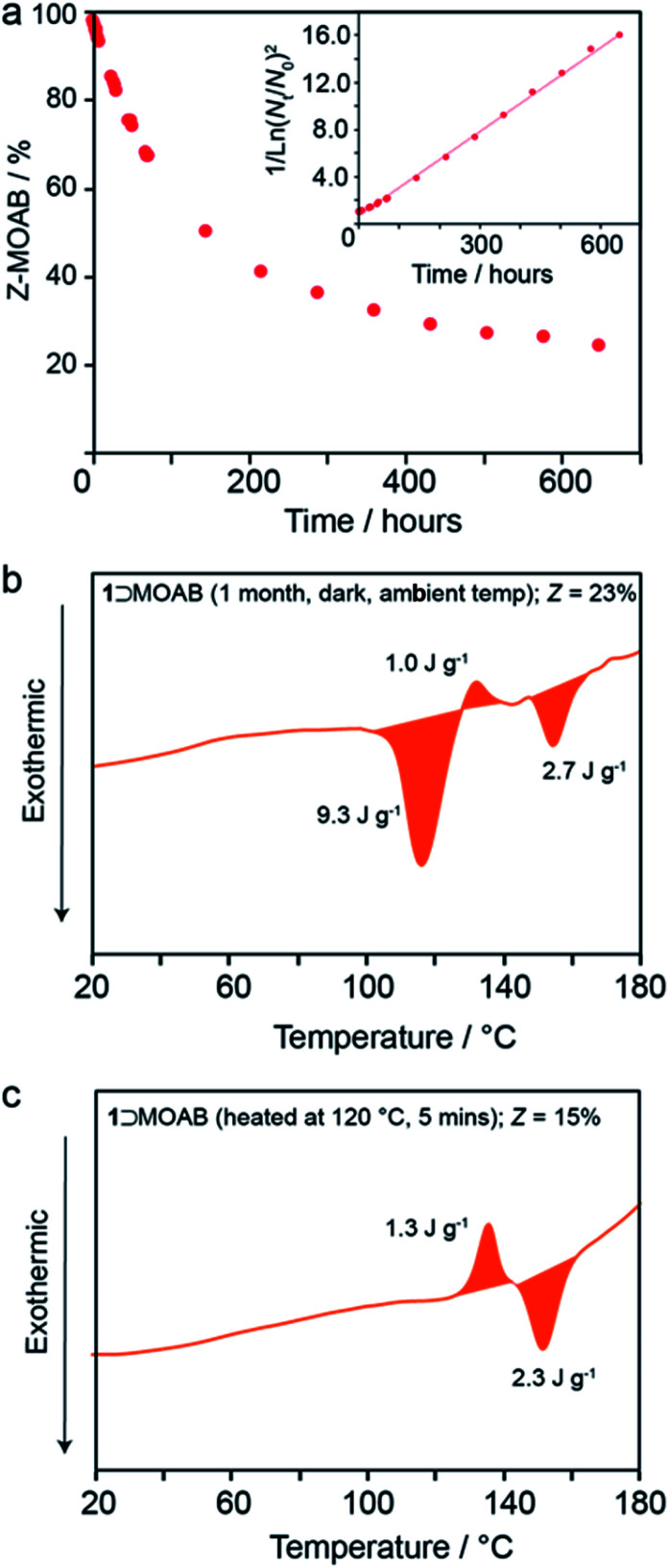
(a) Population of *Z*-MOAB within 1⊃MOAB as a function of time at ambient temperature in the dark and DSC thermograms (first heats) of irradiated 1⊃MOAB after (b) one month in the dark at ambient temperature and (c) 5 minutes at 120 °C.

At ambient temperature in the dark, the occluded *Z*-MOAB molecules thermally reconvert to the *E* isomer with a half-life of approximately 6 days (Fig. 5a and Table S17†). The best fit to the thermal reconversion data was obtained when the process was modelled as following third-order kinetics (Fig. 5a and S34†). This implies a complex cooperative mechanism where motion or rearrangement of multiple *Z*-MOAB molecules is required during the thermal reconversion. This is further supported by DSC thermograms for samples with low *Z*-MOAB populations (Fig. 4b and c), which show complex multicomponent features. The data suggest that there are at least two separate processes characterized by a residual exotherm of 9.3 J g^−1^ (Fig. 5b) and a small composite feature reminiscent of 1⊃AB which remains when the *Z*-MOAB population is further reduced (Fig. 5c).

The 6-day half-life of *Z*-MOAB within the composite is longer than azobenzene-based polymer MOST materials which have been reported in the range 12–75 hours.^45,46^ However, it is significantly shorter than that of *Z*-AB when occluded within 1 (4.5 years).^13^ While the reason for this marked difference requires further investigation, it is likely that the flexible nature of the orthorhombic framework for 1⊃MOAB allows greater freedom of the guest to revert to the *E*-form. This is further supported by the observation by DSC that the thermal reversion begins before the np → lp phase transition in 1⊃MOAB, whereas no thermal reversion is observed in 1⊃AB until the onset of the np → lp phase transition.

## Conclusions

High-efficiency photoswitching in the solid-state remains highly desirable, and the 1⊃MOAB system demonstrates a PSS of >98% for the *Z*-isomer. The composite constitutes a solid-state MOST system that can store 101 J g^−1^ of thermal energy. It also displays a useful half-life of around 6 days at ambient temperature. Reducing the mass of the host MOF or structural modification of the photoswitch could further increase the gravimetric energy density or the half-life of the *Z*-isomer. Both these approaches are currently being targeted and we are also examining the feasibility and efficiency of light-triggered, as well as thermally-triggered, energy release in 1⊃MOAB and other systems.

## Data availability

ESI† for this manuscript can be accessed at DOI: 10.17635/lancaster/researchdata/497.

## Author contributions

All authors conceptualized the research. KG and NH carried out experiments and processed data. The manuscript was written through contributions of all authors. All authors have given approval to the final version of the manuscript.

## Conflicts of interest

There are no conflicts to declare.

## Supplementary Material

SC-013-D2SC00632D-s001
