# Supplementary material for: Efficient solid-state photoswitching of methoxyazobenzene in a metal–organic framework for thermal energy storage
Source: Chem Sci. 2022 Feb 23;13(10):3014–9. doi: 10.1039/d2sc00632d (PMC8905824; doi:10.1039/d2sc00632d)

Electronic Supplementary Material (ESI) for Chemical Science.
This journal is © The Royal Society of Chemistry 2022

# Supporting Information

# Efficient solid-state photoswitching of methoxyazobenzene in a metal-organic framework for thermal energy storage.

Kieran Griffiths[†], Nathan R. Halcovitch[†], John M. Griffin[†‡*]

† Department of Chemistry, Lancaster University, Lancaster, LA1 4YB, UK

‡ Materials Science Institute, Lancaster University, Lancaster, LA1 4YB, UK

Table of Contents

**Experimental Details** ....................4

**Synthesis of 1.** ....................4

**Loading of 1 with MOAB.** ....................4

**Quantification of MOAB loading level by UV-vis spectroscopy** ....................4

**Quantification of loading level by acid digestion and $^1$H NMR spectroscopy.** ....................4

**PSS determination of irradiated 1⊃MOAB composites.** ....................4

**UV light irradiation procedure.** ....................5

**XRPD.** ....................5

**Solid-state NMR.** ....................5

**DFT Calculation Details** ....................5

**Figure S1.** UV-Vis spectra for increasing concentrations of *E*-MOAB in MeOH. ....................7

**Figure S2.** UV-Vis Calibration curve for *E*-MOAB in MeOH. ....................7

**Table S1.** Table for the calibration curve for *E*-MOAB. ....................8

**Table S2.** Determination of the number of moles of *E*-MOAB in **1**⊃MOAB (25mL of MeOH). ....................8

**Table S3.** Determination of the ratio of **1** to *E*-MOAB in **1**⊃MOAB. ....................8

**Figure S3.** DSC thermograms of **1**⊃MOAB and **1**⊃MOAB pre-vacuum treatment. Endothermic transitions below 100 °C are melting transitions for polymorphs of MOAB. These are absent from **1**⊃MOAB. ....................9

**Figure S4.** XRPD comparison for **1**⊃MOAB composites. ....................9

**Figure S5.** Le Bail fit of **1**⊃MOAB. ....................10

**Figure S6.** VT-PXRD patterns for **1**⊃MOAB. ....................10

**Table S4.** Temperature-dependent unit cell lengths for the *Cmmm* (*np*) phase of **1**⊃MOAB, brackets indicate axes converted from *P4/mmm* (lp) phase. ........ 11

**Table S5.** Temperature-dependent unit cell expansion/ contraction for the *Cmmm* (*np*) phase of **1**⊃MOAB, brackets indicate axes converted from *P4/mmm* (*lp*) phase. ........ 11

**Figure S7.** Le Bail fit of **1**⊃MOAB at 40 ........ 11

**Figure S8.** Le Bail fit of **1**⊃MOAB at 60 ........ 12

**Figure S9.** Le Bail fit of **1**⊃MOAB at 80 ........ 12

**Figure S10.** Le Bail fit of **1**⊃MOAB at 100 ........ 12

**Figure S11.** Le Bail fit of **1**⊃MOAB at 120 ........ 13

**Figure S12.** Le Bail fit of **1**⊃MOAB at 140 ........ 13

**Figure S13a.** Le Bail fit of **1**⊃MOAB at 160 °C. ........ 13

**Figure S13b.** Le Bail fit of **1**⊃MOAB at 180 °C. ........ 14

**Figure S14.** $^{13}C$ CPMAS NMR spectra comparing the chemical shifts of **1**, MOAB and of **1**⊃MOAB. ........ 14

**Figure S15.** $^{13}C$ CPMAS NMR spectra of **1**⊃MOAB recorded with different cross-polarisation contact times. ........ 15

**Figure S16.** $^{13}C$ CPMAS NMR spectra of **1**⊃MOAB between 10 – 60 ppm at different MAS frequencies. ........ 16

**Figure S17.** Variable temperature $^{13}C$ CPMAS NMR spectra of **1**⊃MOAB between 110 – 180 ppm. ........ 17

**Table S6**. Calculated chemical shifts for a static molecule of *E*-MOAB. The CASTEP code was used to geometry-optimise a single molecule within a unit cell with fixed dimensions of 10 x 10 x 10 Å before NMR parameters were calculated. ........ 18

**Table S7**. CASTEP calculated chemical shifts for *E*-MOAB assuming fast rotational averaging around the central molecular axis ........ 18

**Figure S18.** Variable-temperature $^{13}C$ CPMAS NMR spectra of **1**⊃MOAB which are compared to the calculated $^{13}C$ chemical shifts of *E*-MOAB. Static and dynamic models for the *E*-MOAB molecule are shown. ........ 19

**Figure S19.** DSC thermogram of **1**⊃MOAB at a rate of 20 °C $min^{-1}$ ........ 20

**Table S8.** Thermal data for the first heating branch of **1**⊃MOAB between 0 – 180 °C at varying heating rates. ........ 20

**Table S9.** Thermal data for the first cooling branch of **1**⊃MOAB between 0 – 180 °C at varying cooling rates. ........ 20

**Figure S20.** Solid-state UV-Vis diffuse reflectance spectra of *E*-MOAB and *Z*-MOAB ........ 21

**Figure S21.** Solid-state UV-Vis diffuse reflectance spectra of **1**, **1**⊃MOAB and irradiated **1**⊃MOAB. ........ 21

**Figure S22.** $^{1}H$ NMR spectra of a) E-MOAB, b) irradiated MOAB (2h), c) MOAB redissolved from **1**⊃MOAB c) before and d) after 540 minutes of irradiation, in benzene-$d_6$. ........ 22

**Figure S23.** Plot of *Z*-MOAB population in benzene-$d_6$ as a function of time following irradiation at 365 nm. ........ 23

**Figure S24.** Plot showing $\ln(N_t/N_0)$ as a function of time, where $N_0$ is the population of *Z*-MOAB immediately after irradiation and $N_t$ is the population of *Z*-MOAB at time *t*. Measurements were

carried out in benzene-$d_6$ solution. The half-life of *Z*-MOAB in benzene solution was determined to be 38.5 hours......23

**Figure S25.** The increase in Z-MOAB proportion when MOAB irradiated in the crystalline state. A plateau is reached at 70% *Z*-MOAB......23

**Figure S26.** DSC thermograms of the first heating branch of **1**⊃MOAB with increasing irradiation time with a scan rate of 20 °C $min^{-1}$......24

**Figure S27.** DSC cycling (irradiation/heat discharge) thermograms for **1**⊃MOAB. The material can be cycled up to five times without a loss in energy density......25

**Table S10.** DFT calculated energy difference between *E*-MOAB and *Z*-MOAB were calculated using the CASTEP code where a single-molecule was confined in a 12 Å x 12 Å x 12 Å cell with fixed unit cell dimensions......26

**Table S11.** Fractional Mass of MOAB in **1**⊃MOAB......26

**Table S12.** Thermal data for the first heating branch **1**⊃MOAB with varying lengths of irradiation. ......27

**Table S13.** Comparison for the experimental energy difference on the first heating branch and the calculated energy difference from *Z* to *E*-MOAB......28

**Figure S28.** Relationship between the experimental energy difference on the first heating branch and the calculated energy difference due to *Z-E* thermal relaxation in **1**⊃MOAB......29

**Figure S29.** Successive DSC thermograms for (top) pristine **1**⊃MOAB (middle) irradiated **1**⊃MOAB and (bottom) thermally reconverted **1**⊃MOAB......29

**Figure S30.** XRPD patterns of irradiated **1**⊃MOAB with specific times under 365 nm light......30

**Figure S31.** Le Bail fit of irradiated **1**⊃MOAB......31

**Table S14.** DFT calculated energy differences between *E* and *Z* isomers of ortho fluorinated azobenzenes (AB-$F_x$) which were reported to be occluded in MOFs......32

**Table S15**. Calculated chemical shifts for a static molecule of *Z*-MOAB. The CASTEP code was used to geometry-optimise a single molecule within a unit cell with fixed dimensions of 12 x 12 x 12 Å before NMR parameters were calculated......33

**Table S16**. CASTEP-calculated chemical shifts for a dynamic molecule of *Z*-MOAB......33

**Figure S32.** Comparison of the DABCO and methoxy regions of $^{13}C$ CPMAS NMR spectra of **1**⊃MOAB and irradiated **1**⊃MOAB......34

**Figure S33.** Comparison of the aromatic regions of $^{13}C$ CPMAS NMR spectra of irradiated **1**⊃MOAB to calculated chemical shifts for *Z*-MOAB......34

**Table S17.** Population of *Z*-MOAB in **1**⊃MOAB measured at different time intervals over a period of one month......35

**Figure S34**. Decay plots for the *Z*-MOAB isomer with respect to order of decay......36

**Experimental Details**

**Synthesis of 1.** All reagents were obtained from Fluorochem and used without further purification. $Zn_2(BDC)_2(DABCO)$ was synthesised according to previously reported synthetic procedures. $Zn(NO_3)_2$•$6H_2O$ (1.68 mmol) was sonicated in N,N-dimethylformamide (DMF, 20 mL) until fully dissolved, 1,4-dibenzendicarboxylic acid (0.28 g, 1.68 mmol) and DABCO (0.093 g, 0.84 mmol) were then added. The reactant solution was placed in a stainless-steel autoclave (Parr) with a Teflon lining with a 50 mL capacity. The solution was heated at 120 °C for 48 h and left to cool to room temperature. The colourless crystals (yield - 81%) were collected via vacuum filtration and washed with DMF (3 x 30 mL) before drying under ambient conditions. Phase purity of the compounds was confirmed by Le Bail fitting of the XRPD patterns.

**Loading of 1 with MOAB.** Samples of **1** were loaded with MOAB using a previously published melt-infiltration procedure. As-prepared samples of **1** were first heated at 120 °C under vacuum for 24 h to remove DMF solvent molecules. 300 mg of the evacuated material was then mixed with a defined mass of *E*-MOAB and heated at 120 °C for 3 h. Excess MOAB was removed by heating at 120 °C under vacuum for 20 h.

**Quantification of MOAB loading level by UV-vis spectroscopy.** UV-vis data was collected on a Cary 60 UV/VIS spectrophotometer with a quartz cell (3 mL) within a 200-600 nm range. A calibration curve with known concentrations of *E*-MOAB was constructed (see Figures S1 and S2).

**Quantification of loading level by acid digestion and $^1$H NMR spectroscopy.** 10 mg of **1**⊃MOAB was suspended in DCl (1.5 mL) and DMSO-$d_6$ (1.5 mL) and placed in a stainless-steel autoclave (Parr) with a Teflon lining with a 50 mL capacity. The suspension was heated for 12 h at 100 °C to yield a transparent solution. A Bruker Avance III 400 NMR spectrometer with a 5 mm $^1$H-X broadband observe probe was used to collected $^1$H NMR data. The $^1$H NMR spectrum of the solution was taken and the ratio between DABCO, BDC and E-MOAB was determined from the integration of characteristic $^1$H resonances.

**PSS determination of irradiated 1⊃MOAB composites.** A 25 mg sample of **1**⊃MOAB was suspended in benzene-$d_6$ (0.5 mL) in an Eppendorf and shaken. The Eppendorf was centrifuged to separate the solid from the solution. A Bruker Avance III 400 NMR spectrometer with a 5 mm $^1$H-X broadband observe probe was used to collect $^1$H NMR data. The population ratio of *E*-MOAB and *Z*-MOAB isomers was determined from integration of *Z*-isomer and *E*-isomer resonances in the $^1$H NMR spectrum based on literature values. The remaining solid was digested in DCl (1.5 mL) and DMSO-$d_6$ (1.5 mL) and placed in a stainless-steel autoclave (Parr) with a Teflon lining with a 50 mL capacity. The suspension was heated for 12 h at 100 °C to yield a transparent solution. The $^1$H NMR spectrum of the solution was taken, and no residual *Z*-MOAB or *E*-MOAB resonances were detected. The process was repeated three separate times and *E/Z* ratios were consistent.

**UV light irradiation procedure.** Samples were irradiated with an OmniCure LX5 LED Head with a power of 425 mW and a 12 mm focusing lens. 50 mg of finely ground **1**⊃MOAB was spread homogenously over a microscope slide. The powder was spread into a circle with a 1 cm radius which was approximately 0.5 mm thick so that irradiation was approximately uniform. The irradiance at central point was 0.3 W $cm^{-2}$. The slide was placed under 365 nm light at a distance of 5 cm. The beam was set to 100% intensity and exposed for a fixed duration. The sample was agitated, at increments of 5 minutes, to allow all particulates to be exposed to the beam.

**XRPD.** X-ray powder diffraction (XRPD) patterns were measured with a Rigaku SmartLab X-ray diffractometer with a 9 kW rotating anode Cu-source equipped with a high-resolution Vertical θ/θ 4-Circle Goniometer and D/teX-ULTRA 250 High-Speed Position-Sensitive Detector System in reflectance mode. The system was configured with parallel-beam optics and a Ge(220) 2 bounce monochromator on the incident side. Powdered solid samples were prepared on glass slides. The measurements were performed as θ/2θ scans with a step size of 0.01 degrees. Variable-temperature measurements were carried out by loading the powder sample into 1 mm diameter thin-walled (0.1mm) borosilicate capillaries, with the capillary loosely sealed with silicone grease to allow for pressure to be released upon heating. The capillary was loaded on a BTS-500 Anton Parr heating stage equipped with a zero-background holder and mounted on a Rigaku SmartLab (9 kW) diffractometer. The heating stage was purged with nitrogen for 30 minutes prior to analysis. The diffractometer was used with parallel-beams optics and a 5 degree soller slit, and a Dtex-250 Ultra 1D detector. The sample run was carried out using a 2-theta scan with a step size of 0.01 degrees 2-theta, and a scanning rate of 4 degrees per minute.

**Solid-state NMR.** Solid-state NMR experiments were performed on Bruker Avance III HD spectrometer operating at magnetic field strength of 16.4 T, corresponding to $^{1}H$ and $^{13}C$ Larmor frequencies of 700 and 176 MHz, respectively. Spectra are referenced relative to tetramethylsilane ($^{13}C$ / $^{1}H$) using the $CH_3$ ($^{1}H$ = 1.1 ppm; $^{13}C$ = 20.5 ppm) resonances of *L*-alanine as a secondary reference. $^{13}C$ NMR spectra were recorded at a magic-angle spinning (MAS) rate of 16.0 kHz using cross polarization (CP) to transfer magnetization from $^{1}H$ with a contact time of 3 ms. The CP pulse was ramped linearly from 70 – 100% power. $^{1}H$ heteronuclear decoupling using two-pulse phase modulation (TPPM)[45] with a pulse length of 4.8 μs and a radiofrequency field strength of 100 kHz was applied during acquisition. Spectra are the sum of 512 transients separated by a recycle interval of 10 s. The sample temperature in variable-temperature experiments was calibrated using $Pb(NO_3)_2$.[46]

**DFT Calculation Details**

First-principles calculations of NMR parameters were carried out under periodic boundary conditons using the CASTEP code[1] employing the gauge-including projector augmented wave (GIPAW) algorithm,[2] which allows the reconstruction of the all-electron wave function in the presence of a magnetic field. The CASTEP calculations employed the generalised gradient approximation Perdew–

Burke–Ernzerhof exchange-correlation functional,[3] and core–valence interactions were described by ultrasoft pseudopotentials.[4]

Prior to calculation of the NMR parameters, structures were fully geometry optimised using the G06 semiempirical dispersion correction scheme[5] and allowing all atomic positions to vary. For calculations on guest-free frameworks, the input atomic co-ordinates were taken from structures published by Dybtsev *et al*., with the guest molecule atoms deleted where appropriate.[6] The tetragonal *lp* structure was based on the empty framework structure, the orthorhombic *np* structure was based on the benzene-loaded structure, and the distorted tetragonal np structure was based on the DMF-loaded structure. These structures also required deletion of some of the atoms representing the dynamic disorder of the DABCO group in order to make a chemically sensible input structure. The structures were then optimized while keeping the unit cell parameters fixed to the experimental values.

Single-molecule calculations were carried out in a 20 × 20 × 20 Å cell with fixed cell parameters to ensure molecules remained isolated from periodic replicas.

Geometry optimisations and NMR calculations were carried out using a planewave energy cut-off of 60 Ry, and for crystal structures, a k-point spacing of 0.05 $2\pi$ Å$^{-1}$ was used. For single-molecule calculations, a single k-point at the fractional coordinate (0.25, 0.25, 0.25) was used. The calculations generate the absolute shielding tensor ($\sigma$) in the crystal frame. Diagonalisation of the symmetric part of $\sigma$ yields the three principal components, $\sigma_{XX}$, $\sigma_{YY}$, and $\sigma_{ZZ}$. The isotropic shielding, $\sigma_{iso}$, is given by (1/3) Tr [$\sigma$]. The iso- tropic chemical shift, $\delta_{iso}$, is given by $\sigma_{ref} - \sigma_{iso}$, where $\sigma_{ref}$ is a reference shielding. Reference shieldings were determined by comparison of experimental chemical shifts for *L*-alanine with shieldings obtained from a calculation on a fully optimised crystal structure[7] (Cambridge Structural Database code LALNIN22). For $^{1}$H and $^{13}$C, reference shieldings were determined from the *y* intercept of a linear fit to the experimental shifts versus calculated shielding, with the gradient of the fit fixed to −1. Calculated shieldings for the three methyl protons were averaged to account for rapid rotation of the methyl group. Respective reference shieldings of 30.2 and 168.4 ppm were obtained for $^{1}$H and $^{13}$C. Calculated chemical shifts for the individual carbons in DABCO groups were averaged to account for the fast rotational dynamics of this group.

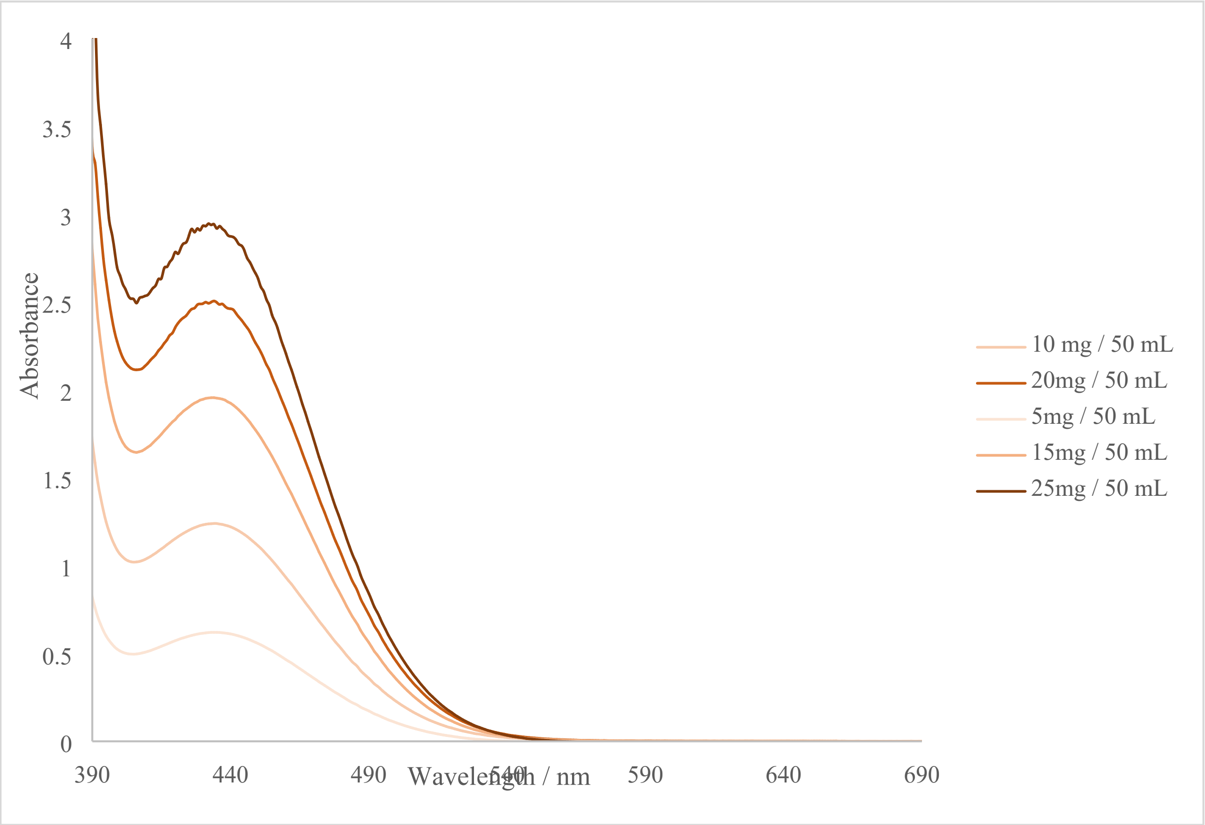


**Figure S1.** UV-Vis spectra for increasing concentrations of *E*-MOAB in MeOH.

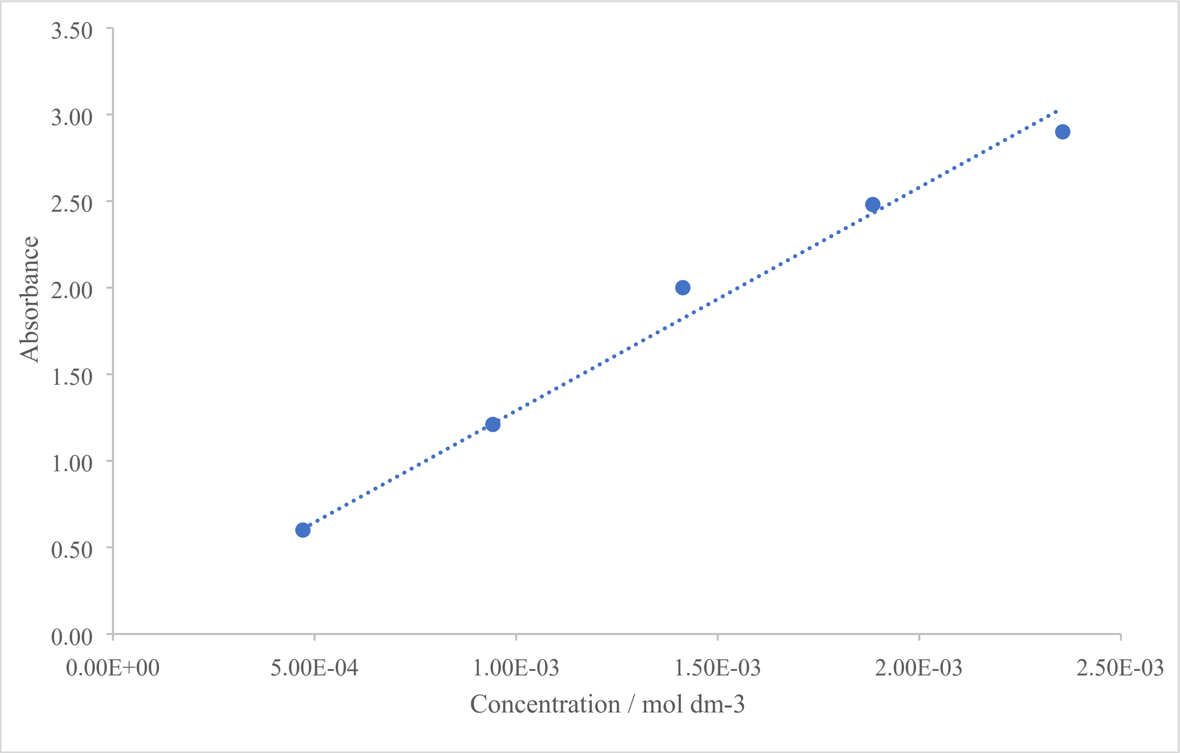


**Figure S2.** UV-Vis Calibration curve for *E*-MOAB in MeOH.

**Table S1.** Table for the calibration curve for *E*-MOAB.

| mass / mg | mass / g | Moles / mol | concentration /mol $dm^{-3}$ | Absorbance |
|---|---|---|---|---|
| 5 | 0.005 | 0.000023557 | 4.71E-04 | 0.60 |
| 10 | 0.01 | 0.000047114 | 9.42E-04 | 1.21 |
| 15 | 0.015 | 0.000070671 | 1.41E-03 | 2.00 |
| 20 | 0.02 | 0.000094229 | 1.88E-03 | 2.48 |
| 25 | 0.025 | 0.000117786 | 2.36E-03 | 2.90 |

**Table S2.** Determination of the number of moles of *E*-MOAB in **1**⊃MOAB (25mL of MeOH).

| Entry | Mass of **1**MOAB / mg | mass / g | Absorbance | Concentration /mol $dm^{-3}$ | Moles / mol | mass of MOAB / g | mass / mg |
|---|---|---|---|---|---|---|---|
| 1 | 19.8 | 0.0198 | 1.53 | 0.001185587 | 0.0000296397 | 0.006291021 | 6.291020922 |
| 2 | 18.7 | 0.0187 | 1.46 | 0.001131344 | 0.0000282836 | 0.006003196 | 6.003196435 |
| 3 | 19.3 | 0.0193 | 1.48 | 0.001146842 | 0.0000286711 | 0.006085432 | 6.085432003 |
| | | | | | | | 6.126549787 |

**Table S3.** Determination of the ratio of **1** to *E*-MOAB in **1**⊃MOAB.

| Entry | Mass of 1 /mg | Mass of **1** / g | mol of **1** / mol | mol of MOAB | Ratio **1** | Ratio MOAB |
|---|---|---|---|---|---|---|
| 1 | 13.50897908 | 0.013508979 | 0.000023650588 | 0.0000296397 | 1 | 1.253232062 |
| 2 | 12.69680356 | 0.012696804 | 0.000022228687 | 0.0000282836 | 1 | 1.272392353 |
| 3 | 13.214568 | 0.013214568 | 0.000023135153 | 0.0000286711 | 1 | 1.239285421 |
| | | | | | | 1.254969945 |

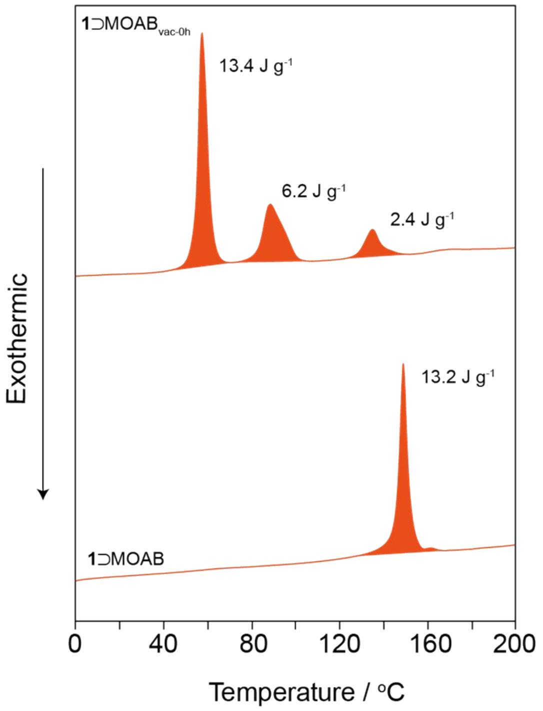


**Figure S3.** DSC thermograms of **1**⊃MOAB and **1**⊃MOAB pre-vacuum treatment. Endothermic transitions below 100 °C are melting transitions for polymorphs of MOAB. These are absent from **1**⊃MOAB.

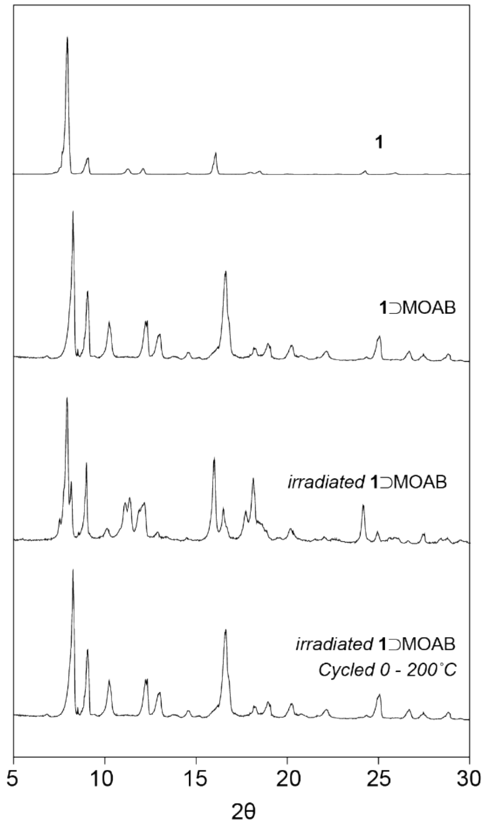


**Figure S4.** XRPD comparison for **1**⊃MOAB composites.

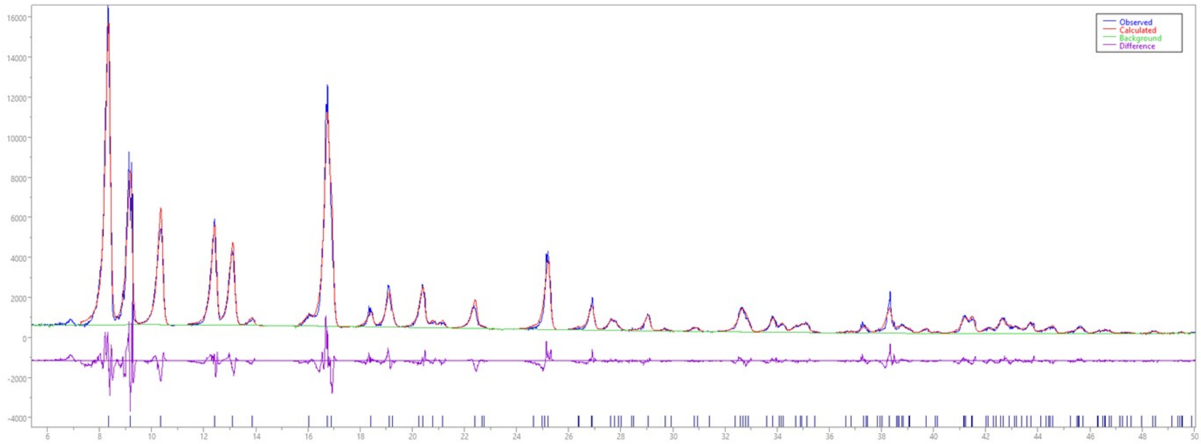


**Figure S5.** Le Bail fit of **1**⊃MOAB. The crystal system was found to be orthorhombic. The lattice parameters were refined to be $a$ =13.492(2) Å, $b$ = 17.129(4), $c$ = 9.679(8) Å, $a$ = $b$ = $g$ = 90°, V = 2236.9(3) Å$^3$. The space group was found to be *Cmmm*. General formula $Zn_8C_{128}H_{120}N_{16}O_{32}$. The reliability (R) factor based on the powder profile *Rp* was 8.65 %.

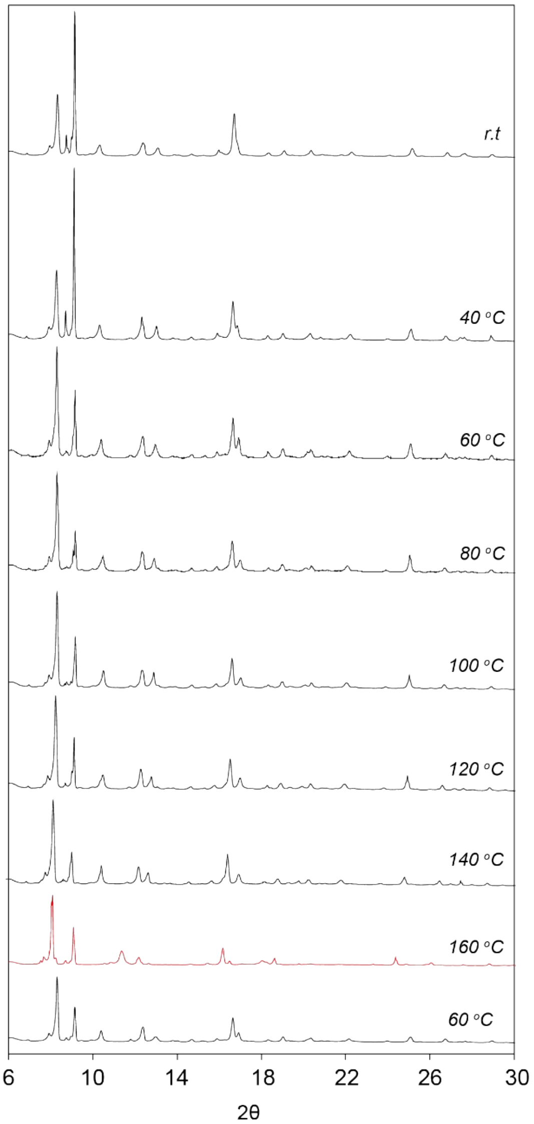


**Figure S6.** VT-PXRD patterns for **1**⊃MOAB.

**Table S4.** Temperature-dependent unit cell lengths for the *Cmmm* (*np*) phase of **1**⊃MOAB, brackets indicate axes converted from *P4/mmm* (lp) phase.

| Temperature / °C | *a* / Å | *b* / Å | *c* / Å | V / Å$^3$ |
|---|---|---|---|---|
| 20 | 13.492 | 17.129 | 9.679 | 2236.9 |
| 40 | 13.566 | 17.105 | 9.681 | 2246.4 |
| 60 | 13.629 | 17.049 | 9.679 | 2249.0 |
| 80 | 13.701 | 16.944 | 9.672 | 2245.4 |
| 100 | 13.754 | 16.855 | 9.681 | 2244.3 |
| 120 | 13.788 | 16.768 | 9.681 | 2238.2 |
| 140 | 13.802 | 16.703 | 9.681 | 2231.8 |
| [160] | [15.501] | [15.501] | [9.706] | [2332.2] |
| [180] | [15.501] | [15.501] | [9.706] | [2332.2] |

**Table S5.** Temperature-dependent unit cell expansion/ contraction for the *Cmmm* (*np*) phase of **1**⊃MOAB, brackets indicate axes converted from *P4/mmm* (*lp*) phase.

| Temperature | *a* expansion / % | *b* contraction / % | *c* / % | V / % |
|---|---|---|---|---|
| 20 | 0.00 | 0.00 | 0.00 | 0.00 |
| 40 | 0.55 | -0.14 | 0.02 | 0.42 |
| 60 | 1.02 | -0.47 | 0.00 | 0.55 |
| 80 | 1.55 | -1.08 | -0.37 | 0.61 |
| 100 | 1.94 | -1.60 | 0.02 | 0.33 |
| 120 | 2.20 | -2.11 | 0.02 | 0.06 |
| 140 | 2.30 | -2.49 | -0.50 | -0.23 |
| [160] | [14.9] | [-9.50] | [0.27] | [4.26] |
| [180] | [14.9] | [-9.50] | [0.27] | [4.26] |

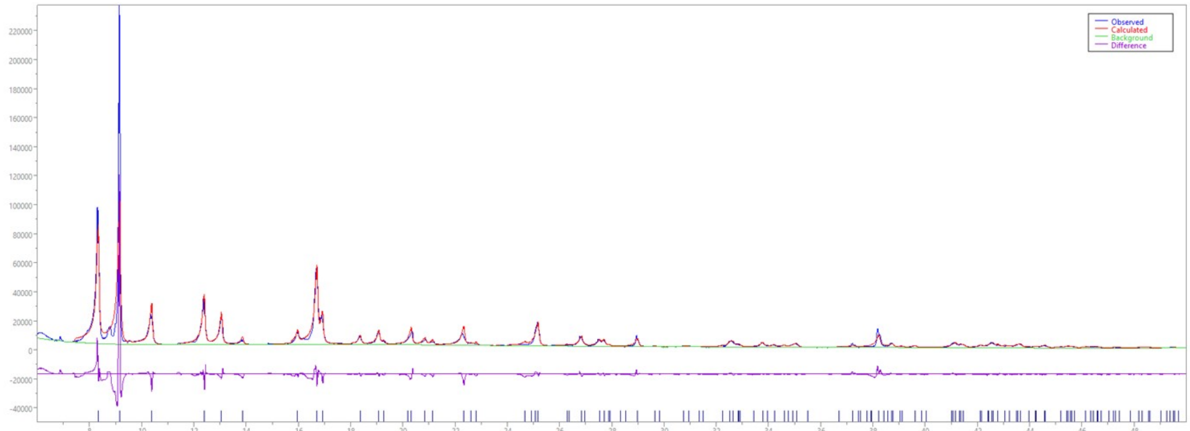


**Figure S7.** Le Bail fit of **1**⊃MOAB at 40 °C. The crystal system was found to be orthorhombic. The lattice parameters were refined to be *a* =13.566(3) Å, *b* = 17.105(2), *c* = 9.681 (7) Å, $\alpha = \beta = \gamma = 90°$, V = 2246.4(4) Å$^3$. The space group was found to be *Cmmm*. General formula $Zn_8C_{128}H_{120}N_{16}O_{32}$. The reliability (R) factor based on the powder profile *Rp* was 8.99 %.

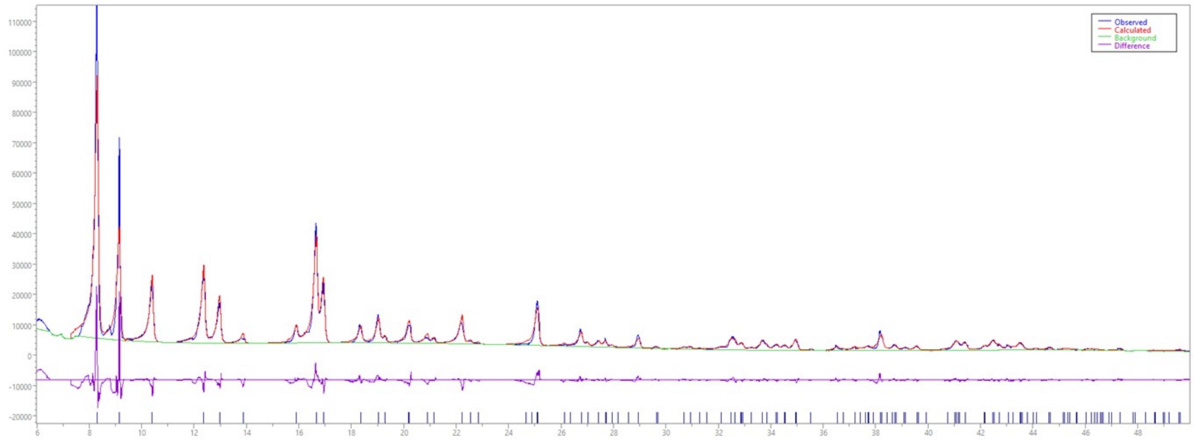


**Figure S8.** Le Bail fit of **1**$\supset$MOAB at 60 °C. The crystal system was found to be orthorhombic. The lattice parameters were refined to be *a* =13.629(4) Å, *b* = 17.049 (3), *c* = 9.679 (5) Å, $\alpha = \beta = \gamma = 90°$, V = 2249.0(4) Å$^3$. The space group was found to be *Cmmm*. General formula $Zn_8C_{128}H_{120}N_{16}O_{32}$. The reliability (R) factor based on the powder profile *Rp* was 8.12 %.

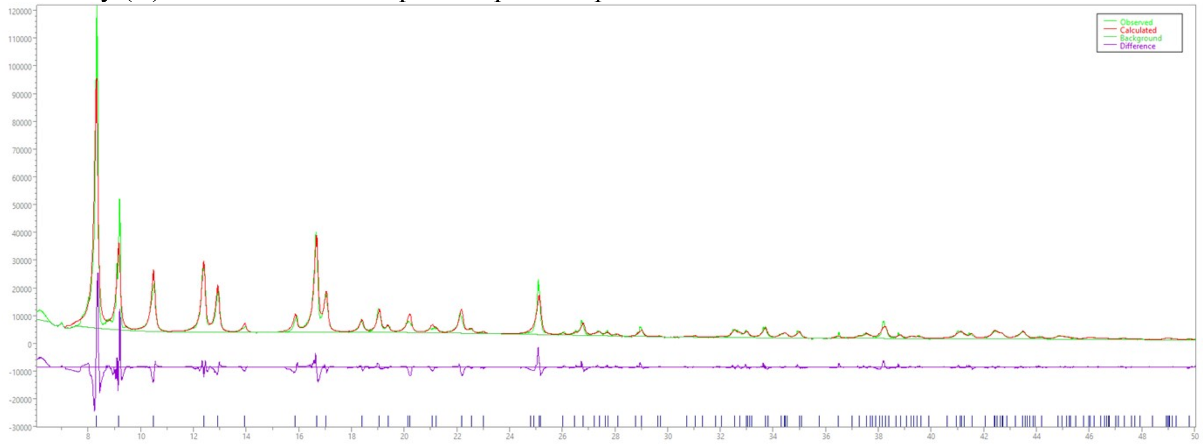


**Figure S9.** Le Bail fit of **1**$\supset$MOAB at 80 °C. The crystal system was found to be orthorhombic. The lattice parameters were refined to be *a* =13.701(2) Å, *b* = 16. 944(3), *c* = 9.672 (5) Å, $\alpha = \beta = \gamma = 90°$, V = 2245.4(4) Å$^3$. The space group was found to be *Cmmm*. General formula $Zn_8C_{128}H_{120}N_{16}O_{32}$. The reliability (R) factor based on the powder profile *Rp* was 7.01 %.

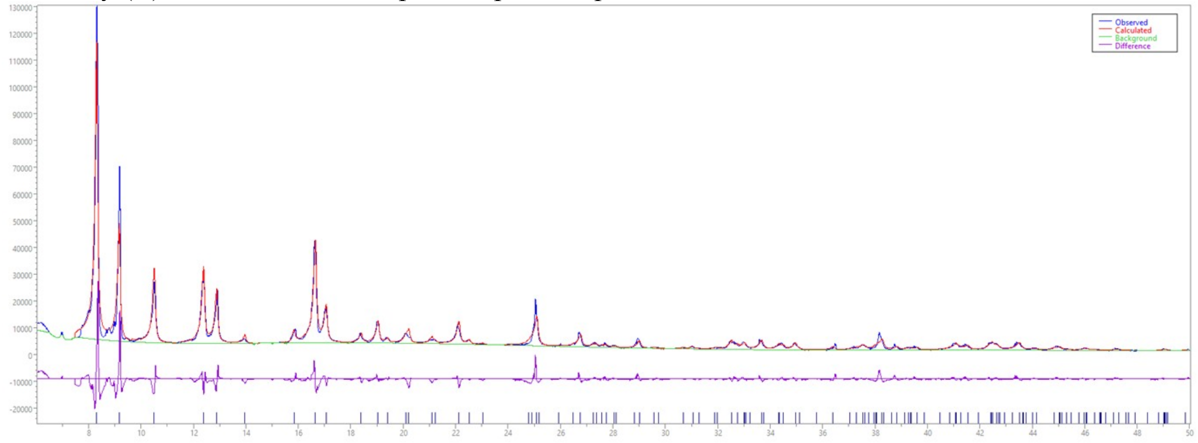


**Figure S10.** Le Bail fit of **1**$\supset$MOAB at 100 °C. The crystal system was found to be orthorhombic. The lattice parameters were refined to be *a* =13.754(3) Å, b = 16.855(3), *c* = 9.681 (6) Å, $\alpha = \beta = \gamma = 90°$, V = 2244.3(4) Å$^3$. The space group was found to be *Cmmm*. General formula $Zn_8C_{128}H_{120}N_{16}O_{32}$. The reliability (R) factor based on the powder profile *Rp* was 5.11 %.

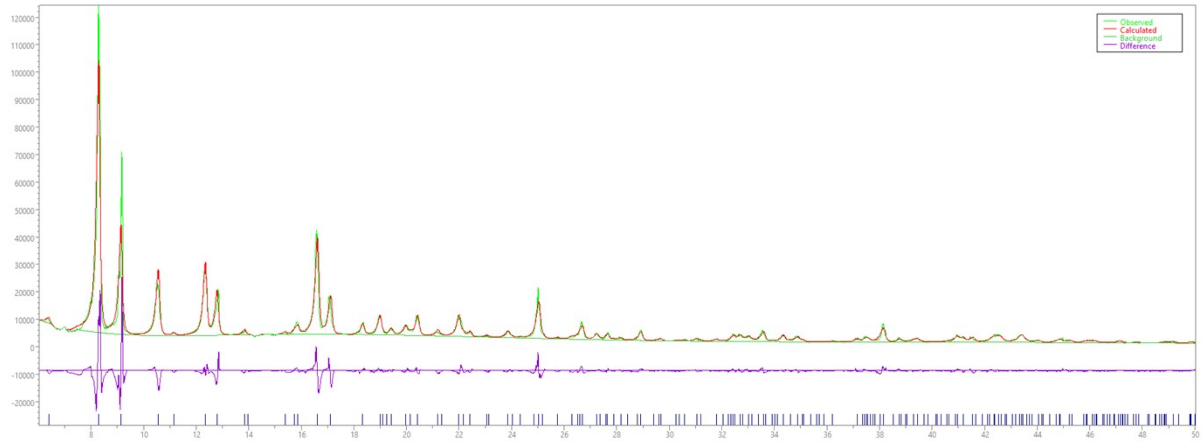


**Figure S11.** Le Bail fit of **1**⊃MOAB at 120 °C. The crystal system was found to be orthorhombic. The lattice parameters were refined to be *a* =13.788(4) Å, b = 16.768(3), *c* = 9.681 (5) Å, $\alpha = \beta = \gamma = 90°$, V = 2240.4(6) Å$^3$. The space group was found to be *Cmmm*. General formula $Zn_8C_{128}H_{120}N_{16}O_{32}$. The reliability (R) factor based on the powder profile *Rp* was 7.95 %.

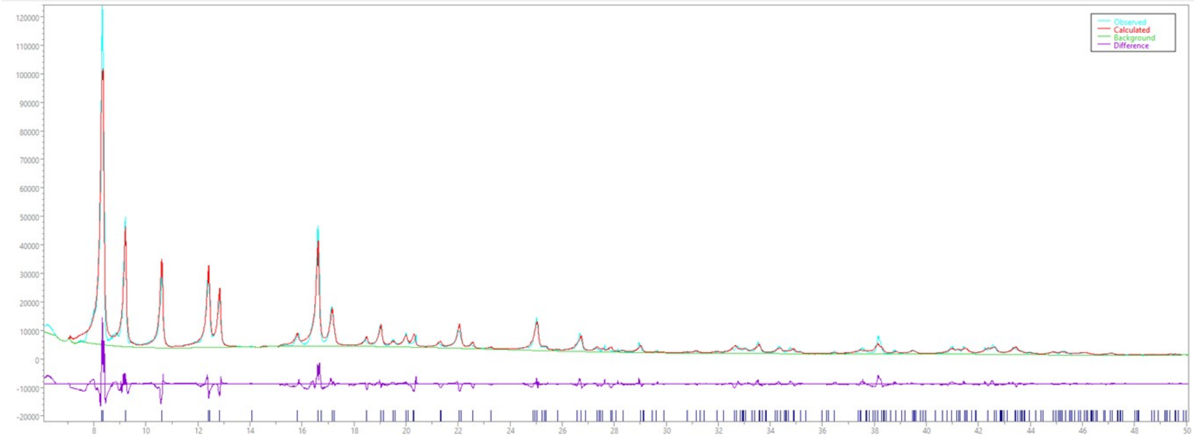


**Figure S12.** Le Bail fit of **1**⊃MOAB at 140 °C. The crystal system was found to be orthorhombic. The lattice parameters were refined to be *a* =13.801(2) Å, *b* = 16.703(2), *c* = 9.681 (4) Å, $\alpha = \beta = \gamma = 90°$, V = 2231.8(8) Å$^3$. The space group was found to be *Cmmm*. General formula $Zn_8C_{128}H_{120}N_{16}O_{32}$. The reliability (R) factor based on the powder profile *Rp* was 6.57 %.

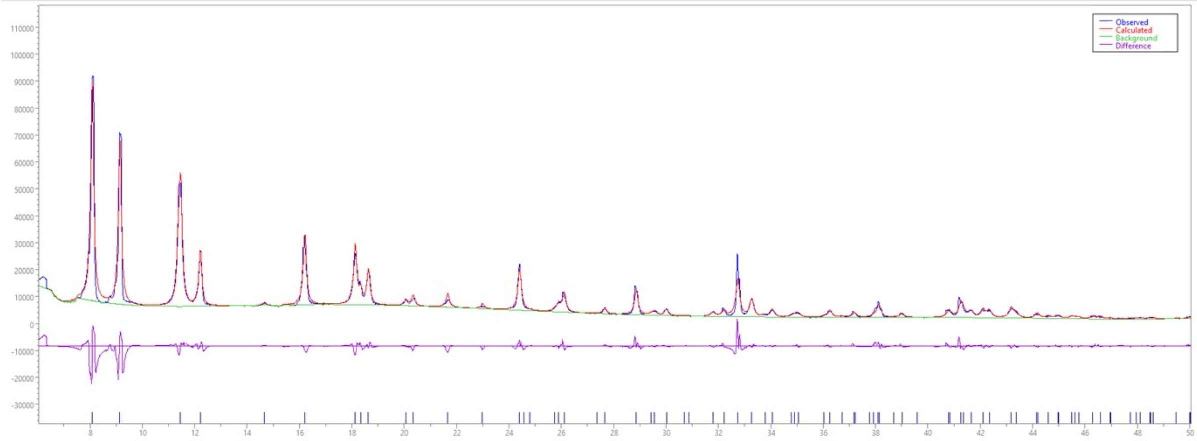


**Figure S13a.** Le Bail fit of **1**⊃MOAB at 160 °C. The crystal system was found to be tetragonal. The lattice parameters were refined to be *a* = *b* = 10.961(3) Å, *c* = 9.706(2) Å, $\alpha = \beta = \gamma = 90°$, V = 1166.12(4) Å$^3$. The space group was found to be *P4/mmm*. General formula $Zn_4C_{64}H_{60}N_8O_{16}$. The reliability (R) factor based on the powder profile *Rp* was 6.95 %.

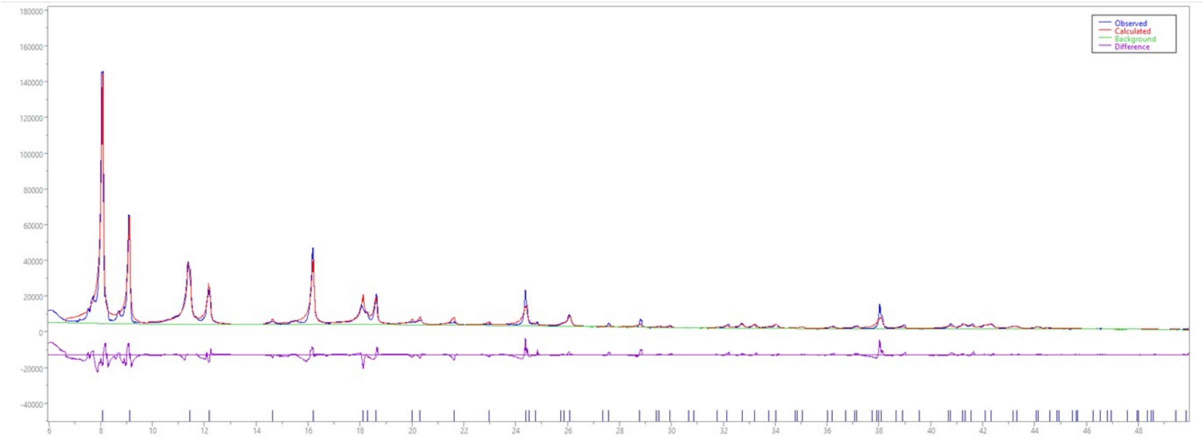


**Figure S13b.** Le Bail fit of **1**⊃MOAB at 180 °C. The crystal system was found to be tetragonal. The lattice parameters were refined to be $a = b = 10.961(4)$ Å, $c = 9.706(5)$ Å, $\alpha = \beta = \gamma = 90°$, V = 1166.12(6) Å$^3$. The space group was found to be *P4/mmm*. General formula $Zn_4C_{64}H_{60}N_8O_{16}$. The reliability (R) factor based on the powder profile *Rp* was 8.91 %.

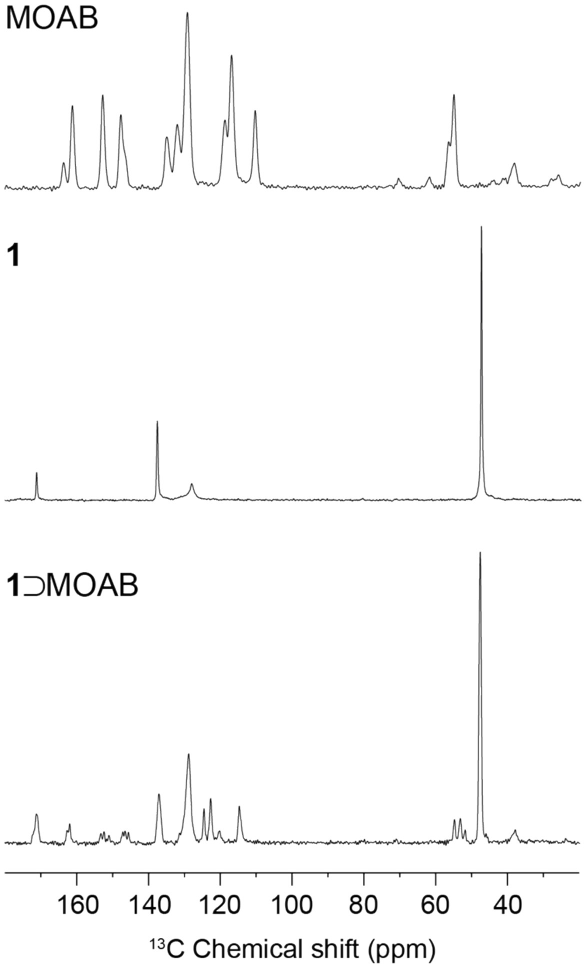


**Figure S14.** $^{13}$C CPMAS NMR spectra comparing the chemical shifts of **1**, MOAB and of **1**⊃MOAB.

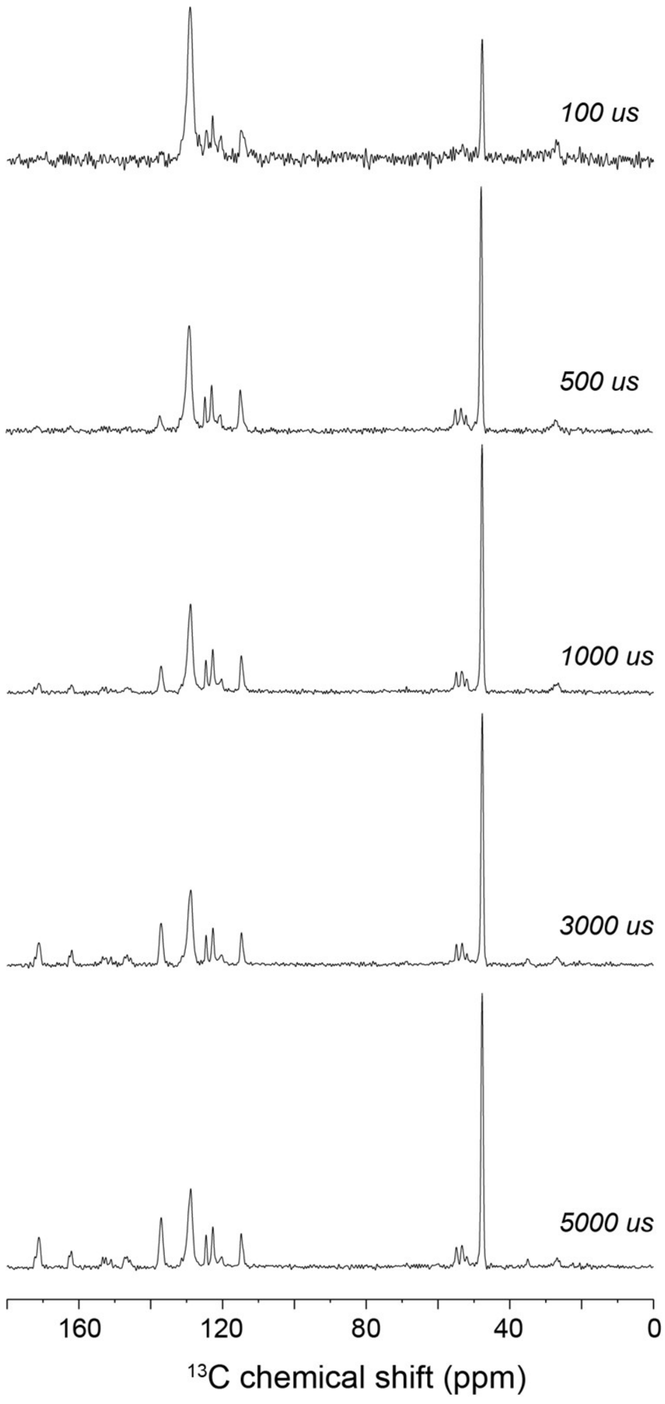


**Figure S15.** $^{13}C$ CPMAS NMR spectra of **1**⊃MOAB recorded with different cross-polarisation contact times.

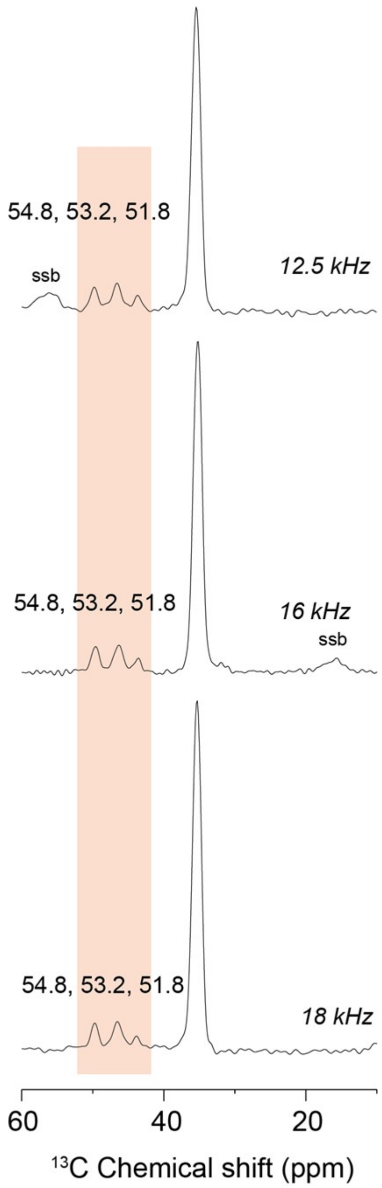


**Figure S16.** $^{13}C$ CPMAS NMR spectra of **1**⊃MOAB between 10 – 60 ppm at different MAS frequencies. This confirms the features seen at 51.8 ppm, 53.2 ppm and 54.8 ppm are from the methoxy carbon of MOAB.

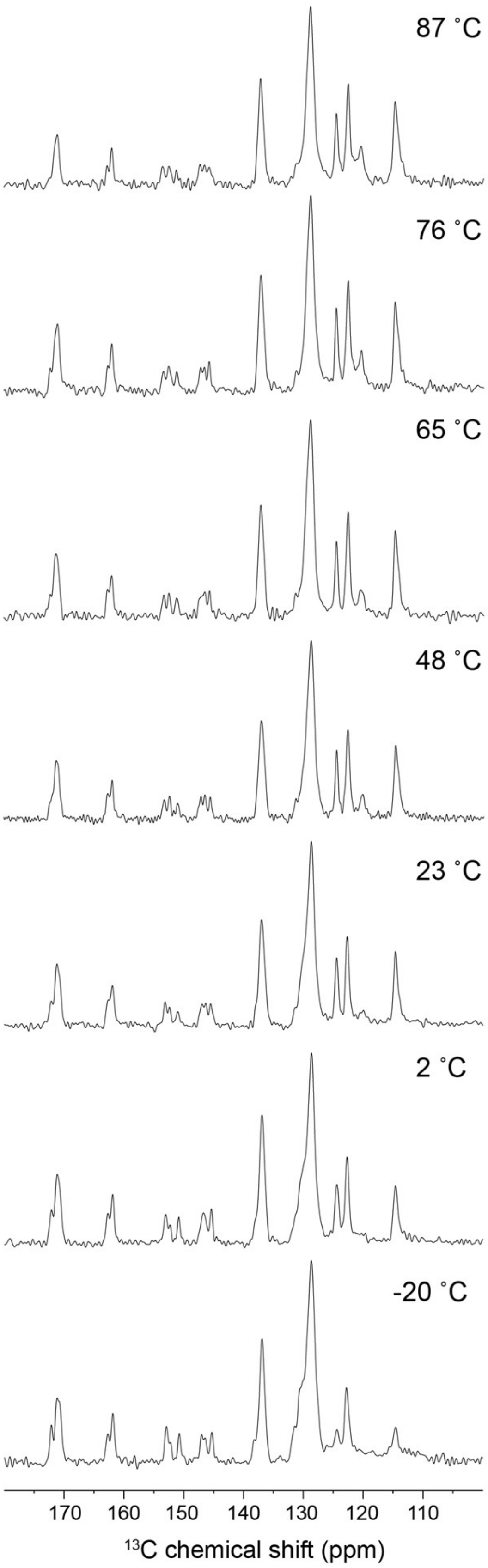

**Figure S17.** Variable temperature $^{13}$C CPMAS NMR spectra of **1**⊃MOAB between 110 – 180 ppm.

**Table S6**. Calculated chemical shifts for a static molecule of *E*-MOAB. The CASTEP code was used to geometry-optimise a single molecule within a unit cell with fixed dimensions of 12 x 12 x 12 Å before NMR parameters were calculated.

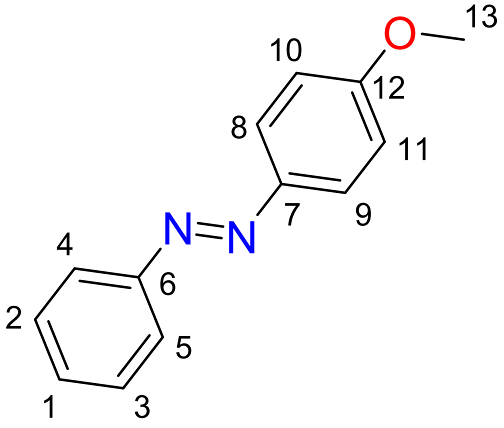


| C site | $\sigma_{iso}$ | Chemical shift / ppm |
|---|---|---|
| 1 | 39.8602 | 128.5998 |
| 2 | 41.931 | 126.529 |
| 3 | 41.4416 | 127.0184 |
| 4 | 58.6355 | 109.8245 |
| 5 | 35.2049 | 133.2551 |
| 6 | 15.6751 | 152.7849 |
| 7 | 21.1183 | 147.3417 |
| 8 | 32.5399 | 135.9201 |
| 9 | 56.5976 | 111.8624 |
| 10 | 63.7801 | 104.6799 |
| 11 | 52.9204 | 115.5396 |
| 12 | 4.8127 | 163.6473 |
| 13 | 118.1348 | 50.3252 |

**Table S7**. CASTEP calculated chemical shifts for *E*-MOAB assuming fast rotational averaging around the central molecular axis

| C site | $\sigma_{iso}$ | Chemical shift / ppm |
|---|---|---|
| C1 | 39.8602 | 128.5998 |
| C2/C3 | 41.6863 | 126.7737 |
| C4/C5 | 46.9202 | 121.5398 |
| C6 | 15.6751 | 152.7849 |
| C7 | 21.1183 | 147.3417 |
| C8/C9 | 44.56875 | 123.89125 |
| C10/ C11 | 58.35025 | 110.10975 |
| C12 | 4.8127 | 163.6473 |
| C13 | 118.1348 | 50.3252 |

Fast rotational averaging was accounted for by averaging chemical shifts for carbons on opposite sides of the six-membered rings. This simulates the effect of fast rotation of the six-membered rings around the C-N bond. In terms of the predicted chemical shifts, this averaging is also equivalent to

fast pedal motion dynamics of the central N=N linkage – indeed rotation of the rings and pedal motion of the N=N linkage cannot be distinguished by this method.

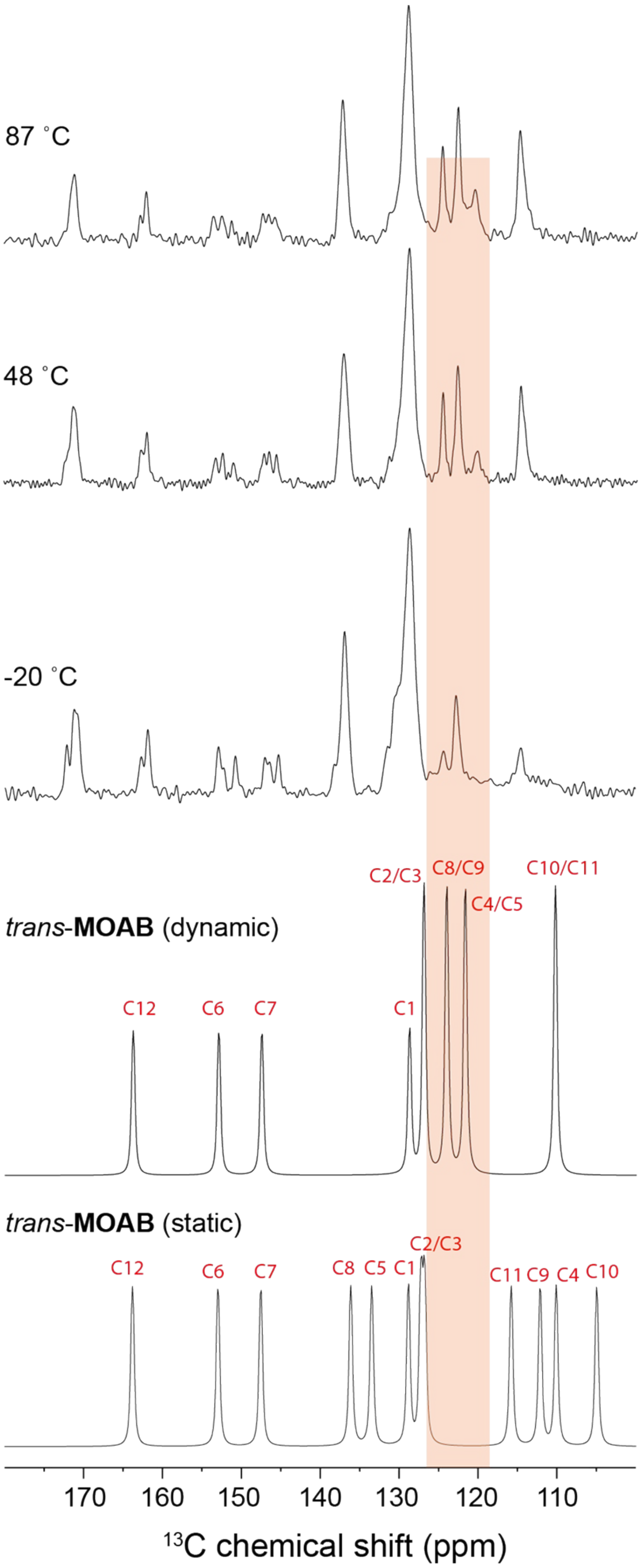


**Figure S18.** Variable-temperature $^{13}$C CPMAS NMR spectra of **1**⊃MOAB which are compared to the calculated $^{13}$C chemical shifts of *E*-MOAB. Static and dynamic models for the *E*-MOAB molecule are shown.

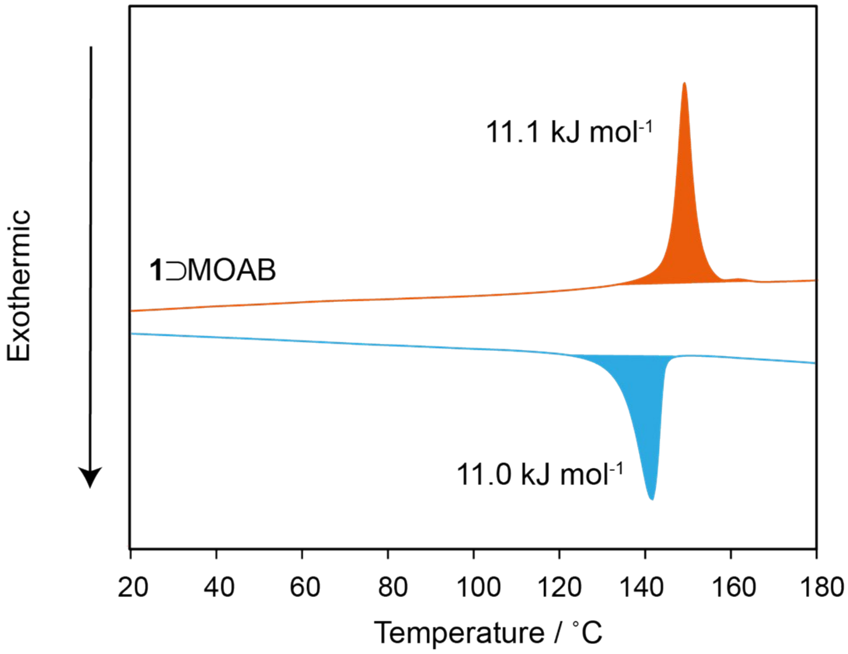


**Figure S19.** DSC thermogram of **1**⊃MOAB at a rate of 20 °C min$^{-1}$.

**Table S8.** Thermal data for the first heating branch of **1**⊃MOAB between 0 – 180 °C at varying heating rates.

| Heating Rate / °C min$^{-1}$ | Energy density/ J g$^{-1}$ | Endotherm onset / °C | Endotherm peak / °C | Peak Range / °C |
|---|---|---|---|---|
| 20 | 13.2 | 145.4 | 149.0 | 38 |
| 15 | 13.1 | 141.8 | 144.8 | 36 |
| 10 | 13.4 | 141.9 | 144.4 | 22 |
| 5 | 13.2 | 141.9 | 144.0 | 20 |
| 2.5 | 13.5 | 142.0 | 143.6 | 20 |

**Table S9.** Thermal data for the first cooling branch of **1**⊃MOAB between 0 – 180 °C at varying cooling rates.

| Cooling Rate / °C min$^{-1}$ | Energy density/ J g$^{-1}$ | Exotherm onset / °C | Exotherm peak / °C | Peak Range / °C |
|---|---|---|---|---|
| 20 | 12.7 | 144.2 | 141.3 | 28 |
| 15 | 13.0 | 141.1 | 138.7 | 35 |
| 10 | 12.2 | 140.9 | 138.8 | 23 |
| 5 | 12.5 | 140.7 | 139.2 | 24 |
| 2.5 | 12.4 | 140.7 | 139.5 | 36 |

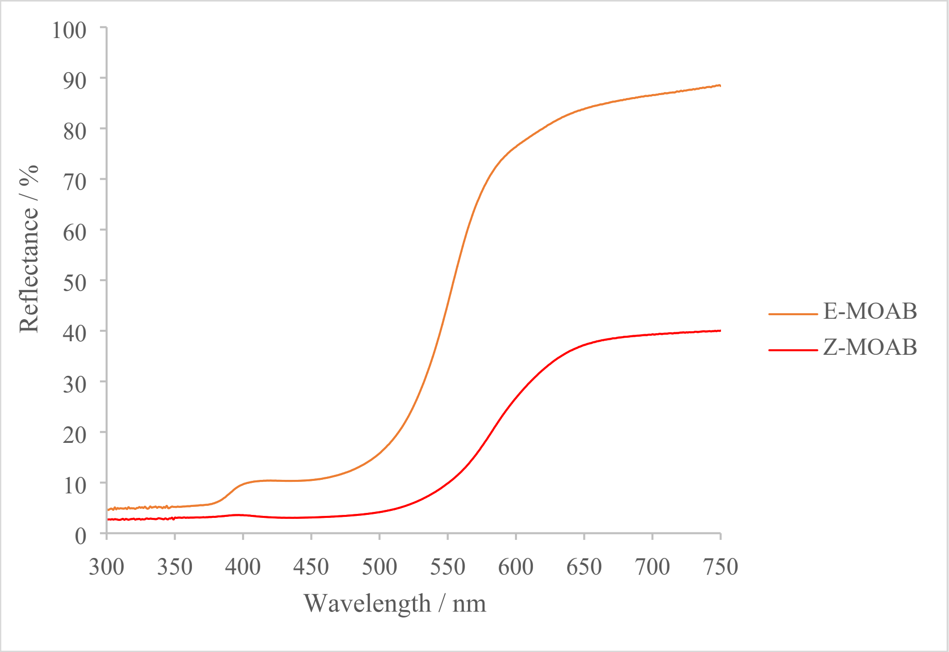


**Figure S20.** Solid-state UV-Vis diffuse reflectance spectra of *E*-MOAB and *Z*-MOAB.

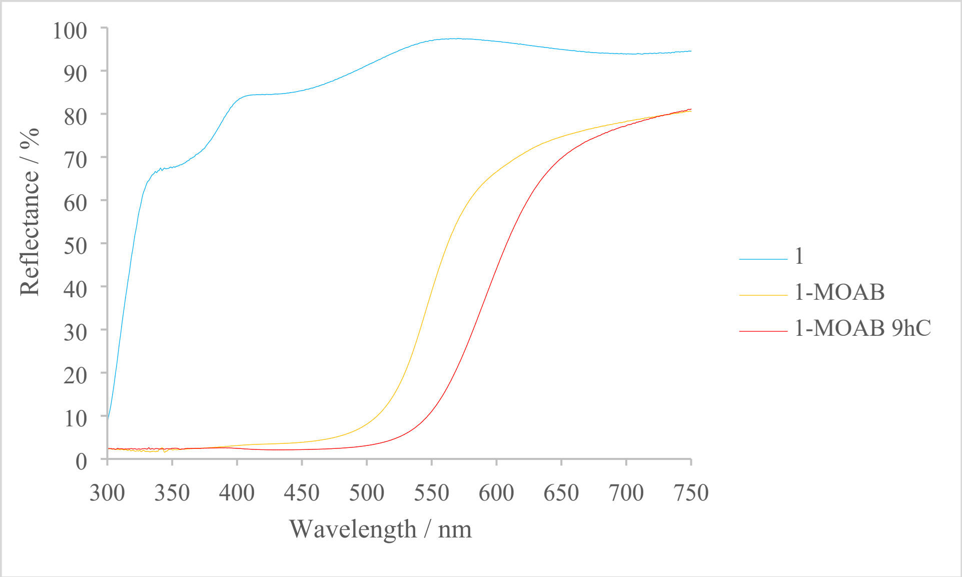


**Figure S21.** Solid-state UV-Vis diffuse reflectance spectra of **1**, **1**⊃MOAB and irradiated **1**⊃MOAB.

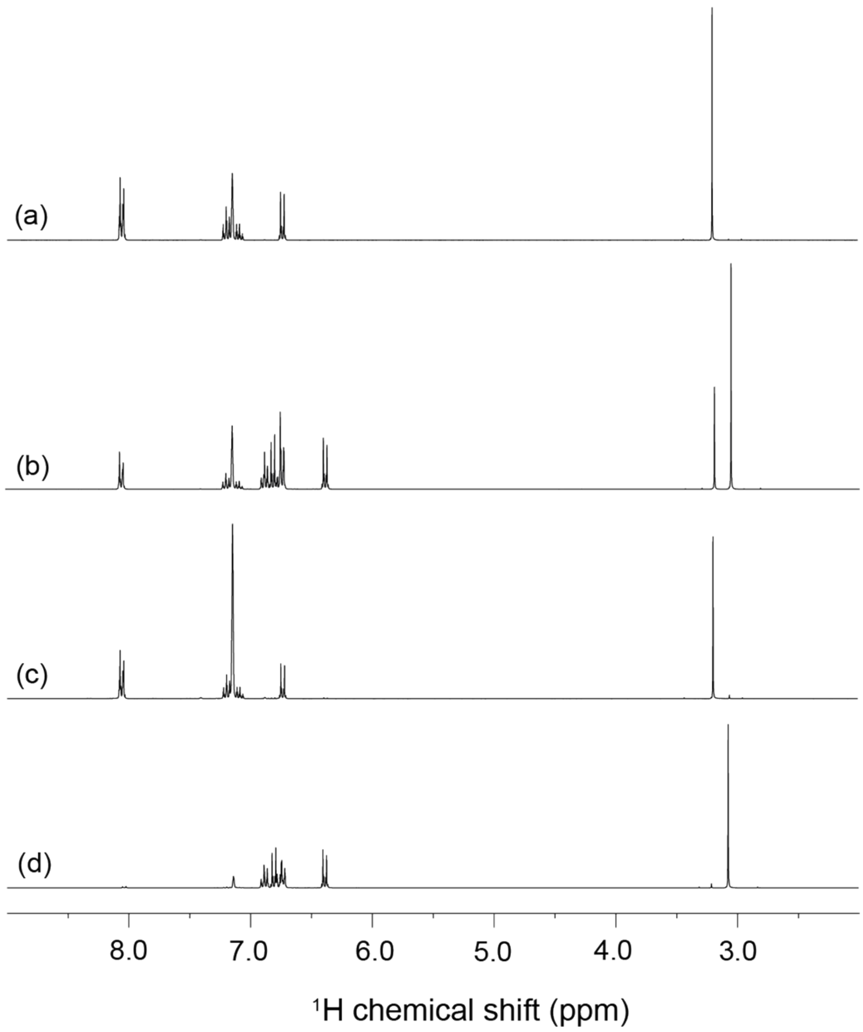


**Figure S22.** $^1$H NMR spectra of a) E-MOAB, b) irradiated MOAB (2h), c) MOAB redissolved from **1**⊃MOAB c) before and d) after 540 minutes of irradiation, in benzene-$d_6$.

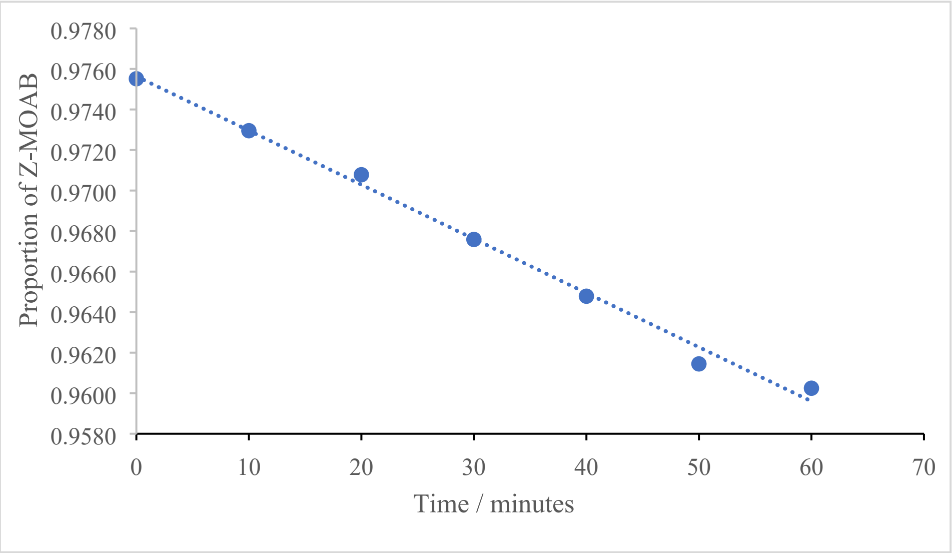

**Figure S23.** Plot of *Z*-MOAB population in benzene-$d_6$ as a function of time following irradiation at 365 nm.

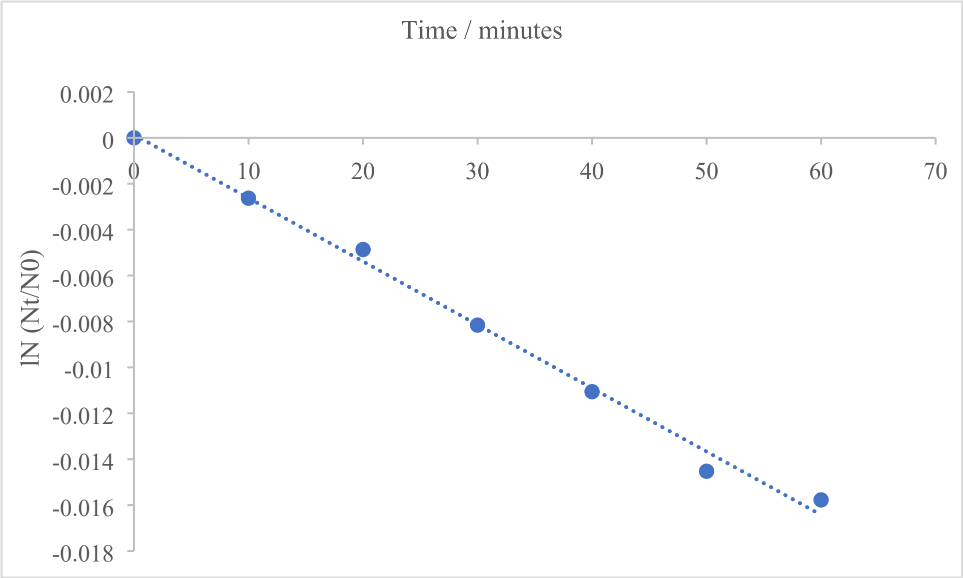


**Figure S24.** Plot showing $\ln(N_t/N_0)$ as a function of time, where $N_0$ is the population of *Z*-MOAB immediately after irradiation and $N_t$ is the population of *Z*-MOAB at time *t*. Measurements were carried out in benzene-$d_6$ solution. The half-life of *Z*-MOAB in benzene solution was determined to be 38.5 hours.

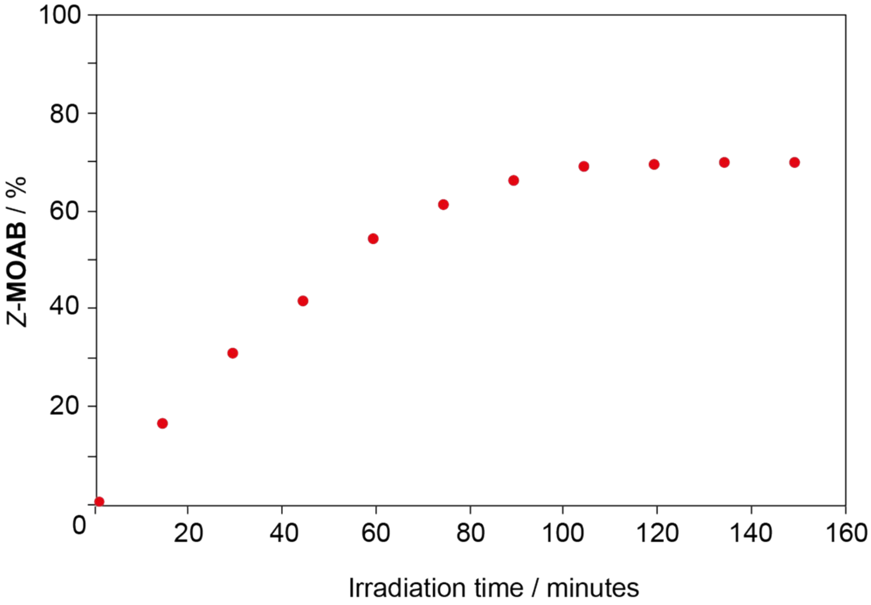


**Figure S25.** The increase in Z-MOAB proportion when MOAB irradiated in the crystalline state. A plateau is reached at 70% *Z*-MOAB.

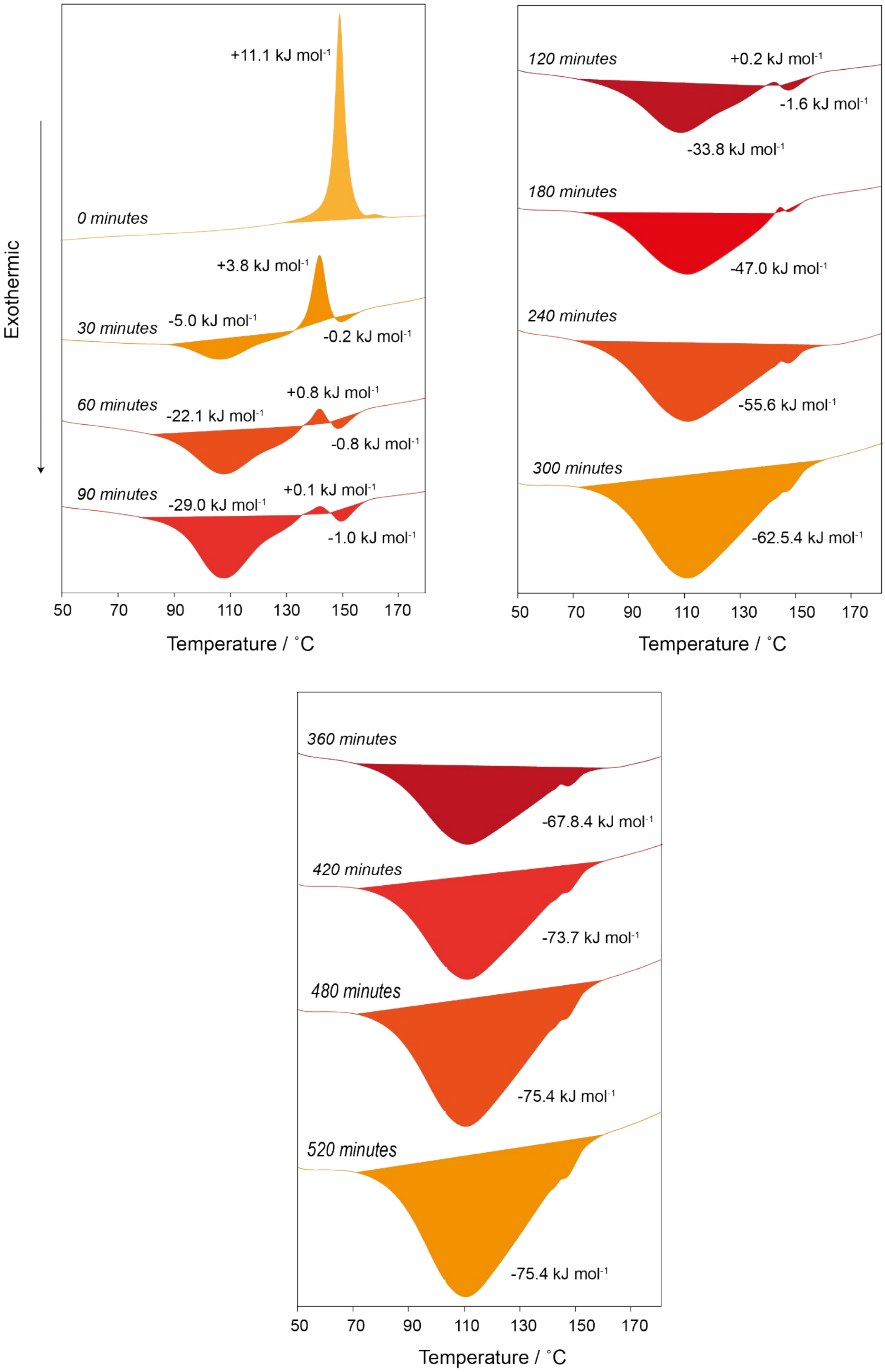


**Figure S26.** DSC thermograms of the first heating branch of **1**⊃MOAB with increasing irradiation time with a scan rate of 20 °C min$^{-1}$.

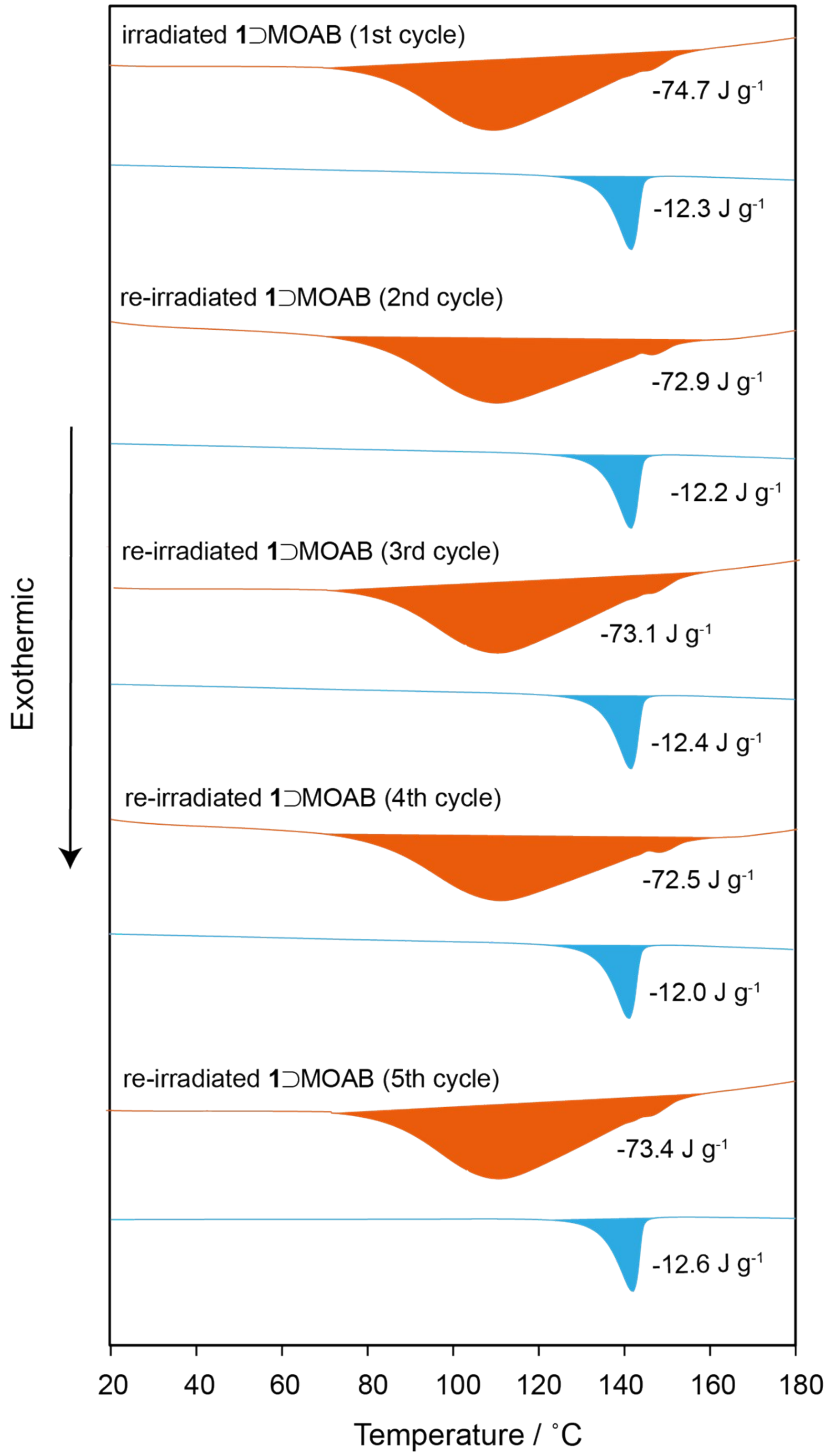


**Figure S27.** DSC cycling (irradiation/heat discharge) thermograms for **1**⊃MOAB. The material can be cycled up to five times without a loss in energy density.

**Table S10.** DFT calculated energy difference between *E*-MOAB and *Z*-MOAB were calculated using the CASTEP code where a single-molecule was confined in a 12 Å x 12 Å x 12 Å cell with fixed unit cell dimensions.

| | Energy / eV molecule$^{-1}$ | Energy / eV mol$^{-1}$ | Energy / J mol$^{-1}$ | Energy Density / J g$^{-1}$ |
|---|---|---|---|---|
| *E*-MOAB | -3233.600200 | -1.9473x10$^{27}$ | -311995649.90 | -1469944.17 |
| *Z*-MOAB | -3232.887900 | -1.9469 x10$^{27}$ | -311926923.25 | -1469620.37 |
| Energy Difference | -0.712300 | -4.2896 x10$^{23}$ | -68726.65 | -323.80 |

**Table S11.** Fractional Mass of MOAB in **1**⊃MOAB.

| Sample | RMM | Fractional mass of MOAB |
|---|---|---|
| **1** | 579.3 | |
| **1**⊃MOAB | 844.6 | 0.31 |

**Table S12.** Thermal data for the first heating branch **1**⊃MOAB with varying lengths of irradiation.

| Time / | Exothermic (1) | Endothermic peak (1)/ J g$^{-1}$ | Exothermic peak (2)/ J g$^{-1}$ | Exothermic peak | Energy difference on heating |
|---|---|---|---|---|---|

| min | / J g$^{-1}$ | | | (3)/ J g-$^{1}$ | branch / J g$^{-1}$ |
|---|---|---|---|---|---|
| 0 | 0.0 | 13.2 | 0 | -12.7 | 0.5 |
| 30 | -5.9 | 4.5 | 0 | -12.6 | -14.1 |
| 60 | -26.2 | 0.9 | -1.01 | -12.5 | -38.8 |
| 120 | -40.0 | 0.2 | -1.88 | -12.5 | -54.2 |
| 180 | -55.6 | 0.0 | -2.62 | -12.6 | -70.8 |
| 240 | -65.9 | 0.0 | 0 | -12.7 | -78.6 |
| 300 | -74.0 | 0.0 | 0 | -12.3 | -86.3 |
| 360 | -80.2 | 0.0 | 0 | -12.8 | -93.0 |
| 420 | -87.2 | 0.0 | 0 | -12.2 | -99.4 |
| 480 | -89.2 | 0.0 | 0 | -12.3 | -101.5 |
| 540 | -89.3 | 0.0 | 0 | -12.5 | -101.8 |

**Table S13.** Comparison for the experimental energy difference on the first heating branch and the calculated energy difference from *Z* to *E*-MOAB.

| Time / min | *Z* / % | Correction for PSS / J g$^{-1}$ | Calculated energy difference / J g$^{-1}$ | Difference between calculated and experimental / J g$^{-1}$ |
|---|---|---|---|---|
| 0 | 0 | 0.00 | 0.00 | 0.50 |

| | | | | |
|---|---|---|---|---|
| 30 | 0.13 | -42.30 | -13.29 | -0.76 |
| 60 | 0.38 | -123.64 | -38.84 | 0.02 |
| 120 | 0.53 | -172.45 | -54.17 | -0.06 |
| 180 | 0.69 | -224.50 | -70.53 | -0.30 |
| 240 | 0.77 | -250.53 | -78.70 | 0.14 |
| 300 | 0.85 | -276.56 | -86.88 | 0.56 |
| 360 | 0.91 | -296.09 | -93.01 | -0.02 |
| 420 | 0.95 | -309.10 | -97.10 | -2.33 |
| 480 | 0.99 | -322.12 | -101.19 | -0.34 |
| 540 | 0.99 | -322.12 | -101.19 | -0.64 |

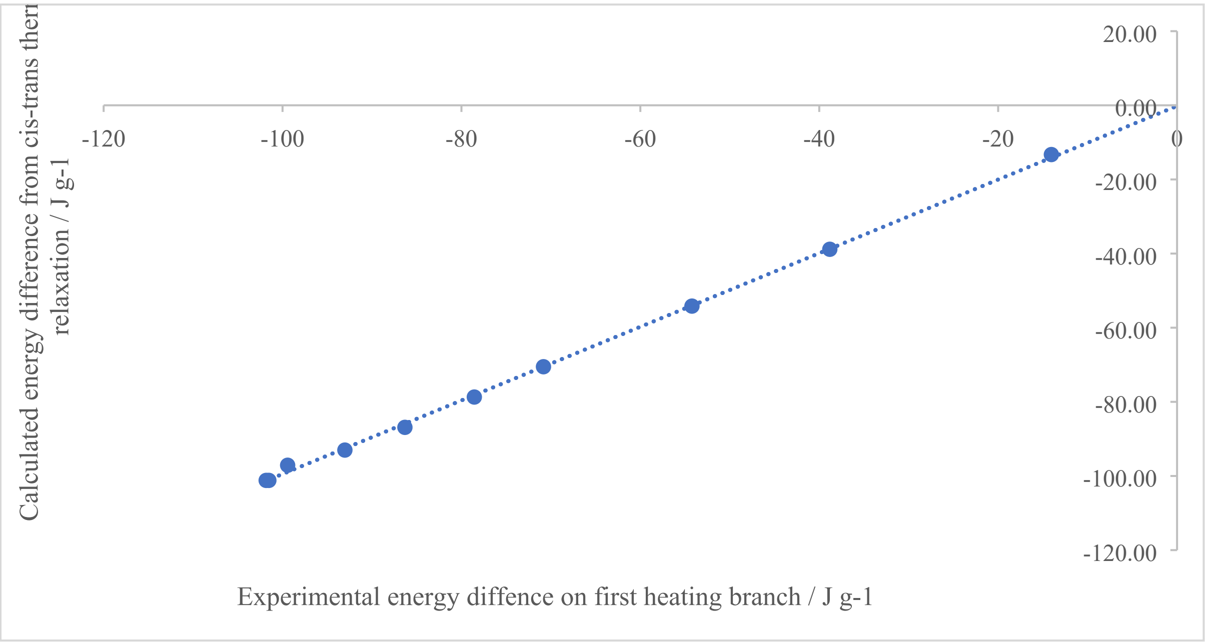


**Figure S28.** Relationship between the experimental energy difference on the first heating branch and the calculated energy difference due to *Z-E* thermal relaxation in **1**⊃MOAB.

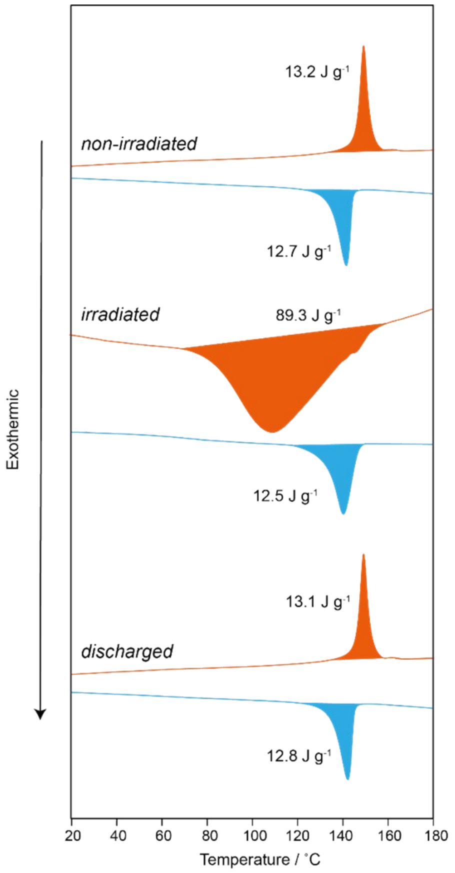


**Figure S29.** Successive DSC thermograms for (top) pristine **1**⊃MOAB (middle) irradiated **1**⊃MOAB and (bottom) thermally reconverted **1**⊃MOAB.

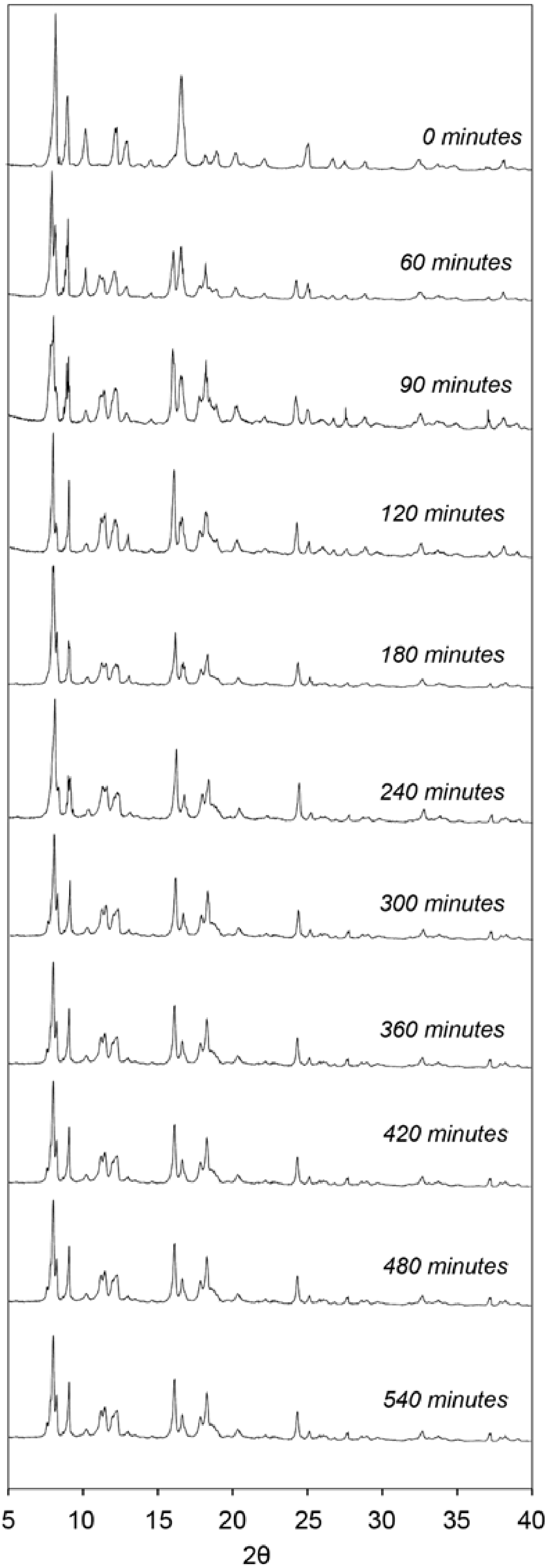


**Figure S30.** XRPD patterns of irradiated **1**⊃MOAB with specific times under 365 nm light.

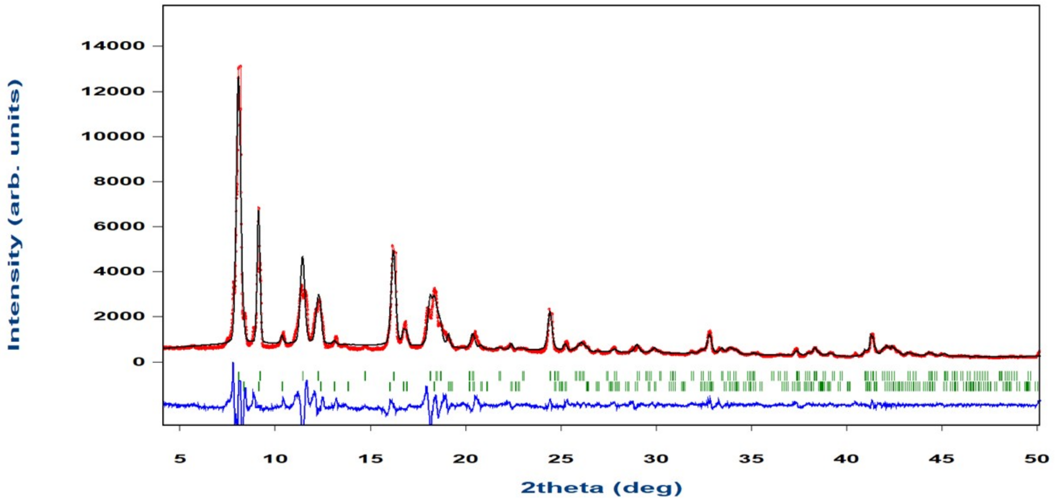


**Figure S31.** Le Bail fit of irradiated **1**⊃MOAB. Two phases were identified and the dominant phase's crystal system was found to be tetragonal. The lattice parameters were refined to be $a = b = 10.982(7)$ Å, $c = 9.619(3)$ Å, $\alpha = \beta = \gamma = 90°$, V = 1169.1(2) Å$^3$. The space group was found to be *P*4*/mmm*. The second crystal system was found to be orthorhombic. The lattice parameters were refined to be $a = 13.492(2)$ Å, $b = 17.129(4)$, $c = 9.679(8)$ Å, $\alpha = \beta = \gamma = 90°$, V = 2236.7(3) Å$^3$. The space group was found to be *Cmmm.* Which is consistent with the pre-irradiated phase. General formula $Zn_8C_{128}H_{120}N_{16}O_{32}$. The reliability (R) factor based on the powder profile *Rp* was 11.19 %.

**Table S14.** DFT calculated energy differences between *E* and *Z* isomers of ortho fluorinated azobenzenes (AB-$F_x$) which were reported to be occluded in MOFs.

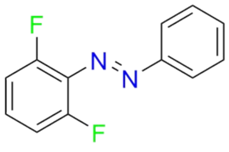

AB-$F_2$

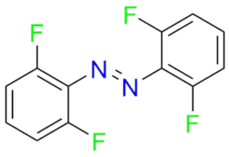

AB-$F_4$

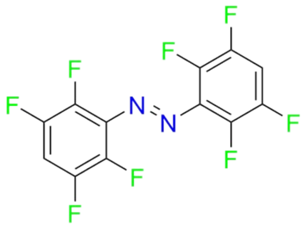

AB-$F_8$

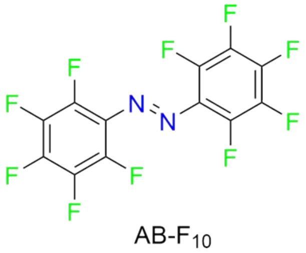

AB-$F_{10}$

| AB-$F_2$ | (energy/molecule) / (eV/molecule) | (energy/mole) / (eV/mol) | (energy/mol) / (J/mol) | kJ / mol |
|---|---|---|---|---|
| *E* isomer | -3894.29 | -2.35E+27 | -375743017.66 | |
| *Z*-isomer | -3893.92 | -2.34E+27 | -375707122.46 | |
| ED | -0.37 | -2.24E+23 | -35895.20 | -35.90 |

| AB-$F_4$ | (energy/molecule) / (eV/molecule) | (energy/mole) / (eV/mol) | (energy/mol) / (J/mol) | kJ / mol |
|---|---|---|---|---|
| *E* isomer | -5184.86 | -3.12E+27 | -500264405.56 | |
| *Z*-isomer | -5184.63 | -3.12E+27 | -500242155.10 | |
| ED | -0.23 | -1.39E+23 | -22250.46 | -22.25 |

| AB-$F_8$ | (energy/molecule) / (eV/molecule) | (energy/mole) / (eV/mol) | (energy/mol) / (J/mol) | kJ / mol |
|---|---|---|---|---|
| *E* isomer | -7765.80 | -4.68E+27 | -749287567.35 | |
| *Z*-isomer | -7765.48 | -4.68E+27 | -749256674.16 | |
| ED | -0.32 | -1.93E+23 | -30893.19 | -30.89 |

| AB-$F_{10}$ | (energy/molecule) / (eV/molecule) | (energy/mole) / (eV/mol) | (energy/mol) / (J/mol) | kJ / mol |
|---|---|---|---|---|
| *E* isomer | -9055.96 | -5.45E+27 | -873769602.97 | |
| *Z*-isomer | -9055.59 | -5.45E+27 | -873733471.02 | |
| ED | -0.37 | -2.26E+23 | -36131.96 | -36.13 |

**Table S15**. Calculated chemical shifts for a static molecule of *Z*-MOAB. The CASTEP code was used to geometry-optimise a single molecule within a unit cell with fixed dimensions of 12 x 12 x 12 Å before NMR parameters were calculated.

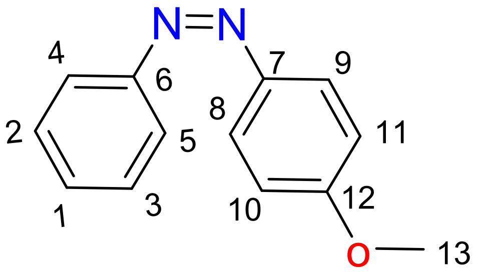


| C site | $\sigma_{iso}$ | Chemical shift / ppm |
|---|---|---|
| C1 | 44.6931 | 123.7669 |
| C2 | 42.7124 | 125.7476 |
| C3 | 40.1623 | 128.2977 |
| C4 | 57.6192 | 110.8408 |
| C5 | 44.2558 | 124.2042 |
| C6 | 11.1773 | 157.2827 |
| C7 | 21.865 | 146.595 |
| C8 | 36.3859 | 132.0741 |
| C9 | 53.4647 | 114.9953 |
| C10 | 62.2751 | 106.1849 |
| C11 | 55.6696 | 112.7904 |
| C12 | 7.8567 | 160.6033 |
| C13 | 117.8611 | 50.5989 |

**Table S16**. CASTEP-calculated chemical shifts for a dynamic molecule of *Z*-MOAB.

| C site | $\sigma_{iso}$ | Chemical shift / ppm |
|---|---|---|
| C1 | 44.6931 | 123.7669 |
| C2/C3 | 41.43735 | 127.02265 |
| C4/C5 | 50.9375 | 117.5225 |
| C6 | 11.1773 | 157.2827 |
| C7 | 21.865 | 146.595 |
| C8/C9 | 44.9253 | 123.5347 |
| C10/C11 | 58.97235 | 109.48765 |
| C12 | 7.8567 | 160.6033 |
| C13 | 117.8611 | 50.5989 |

Fast rotational averaging was accounted for by averaging chemical shifts for carbons on opposite sides of the six-membered rings. This simulates the effect of fast rotation of the six-membered rings around the C-N bond. In terms of the predicted chemical shifts, this averaging is also equivalent to fast pedal motion dynamics of the central N=N linkage – indeed rotation of the rings and pedal motion of the N=N linkage cannot be distinguished by this method.

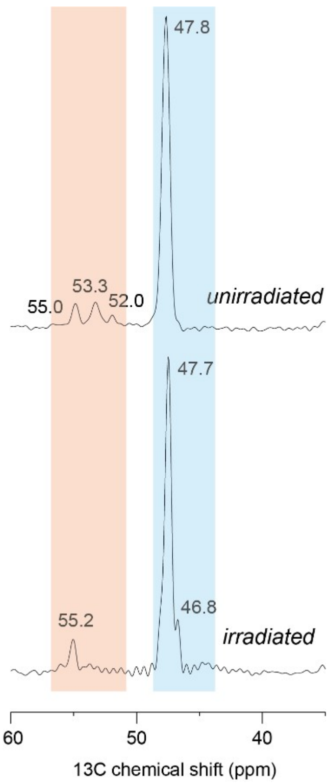


**Figure S32.** Comparison of the DABCO and methoxy regions of $^{13}$C CPMAS NMR spectra of **1**⊃MOAB and irradiated **1**⊃MOAB.

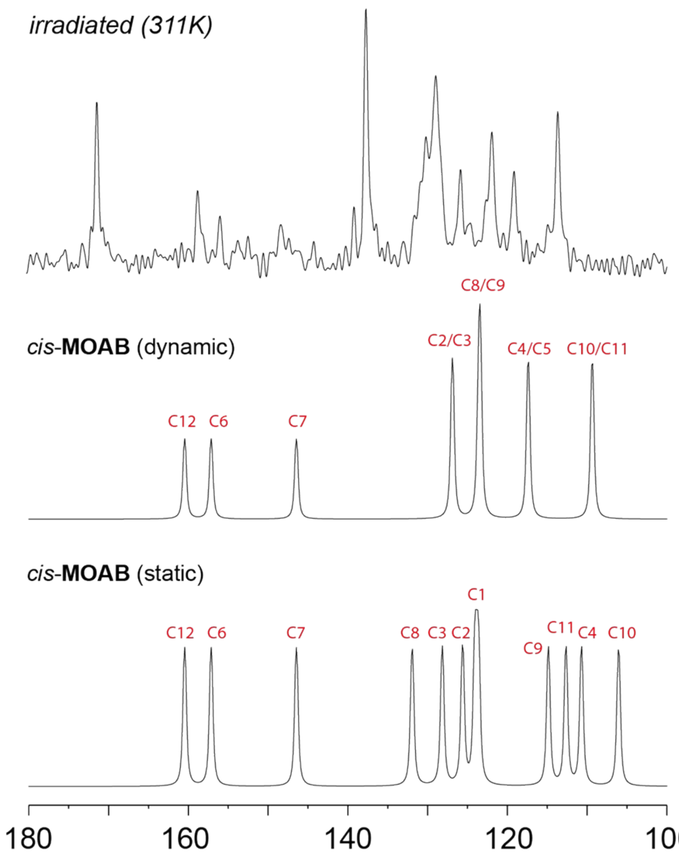


**Figure S33.** Comparison of the aromatic regions of $^{13}$C CPMAS NMR spectra of irradiated **1**⊃MOAB to calculated chemical shifts for *Z*-MOAB.

**Table S17.** Population of *Z*-MOAB in **1**⊃MOAB measured at different time intervals over a period of one month.

| hour | day | Fraction of Z-MOAB | Nt/N0 | Ln(Nt/N0) | 1/(Nt/N0) | 1/(Nt/N0)1.5 | 1/(Nt/N0)2 |
|---|---|---|---|---|---|---|---|
| 0.00 | 0.00 | 0.98 | 1.00 | 0.00 | 1.00 | 1.00 | 1.00 |
| 1.00 | 0.04 | 0.97 | 0.99 | -0.01 | 1.01 | 1.02 | 1.02 |
| 2.00 | 0.08 | 0.97 | 0.99 | -0.01 | 1.01 | 1.02 | 1.02 |
| 3.00 | 0.13 | 0.96 | 0.98 | -0.02 | 1.02 | 1.03 | 1.04 |
| 4.00 | 0.17 | 0.96 | 0.98 | -0.02 | 1.02 | 1.03 | 1.04 |
| 5.00 | 0.21 | 0.95 | 0.97 | -0.03 | 1.03 | 1.05 | 1.06 |
| 6.00 | 0.25 | 0.94 | 0.96 | -0.04 | 1.04 | 1.06 | 1.09 |
| 7.00 | 0.29 | 0.94 | 0.96 | -0.04 | 1.04 | 1.06 | 1.09 |
| 8.00 | 0.33 | 0.93 | 0.95 | -0.05 | 1.05 | 1.08 | 1.11 |
| 24.00 | 1.00 | 0.85 | 0.87 | -0.14 | 1.15 | 1.23 | 1.32 |
| 26.00 | 1.08 | 0.84 | 0.86 | -0.15 | 1.16 | 1.25 | 1.35 |
| 28.00 | 1.17 | 0.83 | 0.85 | -0.16 | 1.18 | 1.28 | 1.38 |
| 30.00 | 1.25 | 0.82 | 0.84 | -0.17 | 1.19 | 1.30 | 1.42 |
| 46.00 | 1.92 | 0.75 | 0.77 | -0.26 | 1.30 | 1.48 | 1.69 |
| 48.00 | 2.00 | 0.75 | 0.76 | -0.27 | 1.32 | 1.51 | 1.73 |
| 50.00 | 2.08 | 0.74 | 0.75 | -0.29 | 1.33 | 1.54 | 1.78 |
| 68.00 | 2.83 | 0.68 | 0.69 | -0.37 | 1.45 | 1.74 | 2.10 |
| 69.00 | 2.88 | 0.67 | 0.69 | -0.37 | 1.45 | 1.74 | 2.10 |
| 70.00 | 2.92 | 0.67 | 0.68 | -0.39 | 1.47 | 1.78 | 2.16 |
| 71.00 | 2.96 | 0.67 | 0.68 | -0.39 | 1.47 | 1.78 | 2.16 |
| 144.00 | 6.00 | 0.50 | 0.51 | -0.67 | 1.96 | 2.75 | 3.84 |
| 216.00 | 9.00 | 0.41 | 0.42 | -0.87 | 2.38 | 3.67 | 5.67 |
| 288.00 | 12.00 | 0.36 | 0.37 | -0.99 | 2.70 | 4.44 | 7.30 |
| 360.00 | 15.00 | 0.32 | 0.33 | -1.11 | 3.03 | 5.28 | 9.18 |
| 432.00 | 18.00 | 0.29 | 0.30 | -1.20 | 3.33 | 6.09 | 11.11 |
| 504.00 | 21.00 | 0.27 | 0.28 | -1.27 | 3.57 | 6.75 | 12.76 |
| 576.00 | 24.00 | 0.26 | 0.26 | -1.35 | 3.85 | 7.54 | 14.79 |
| 648.00 | 27.00 | 0.24 | 0.25 | -1.39 | 4.00 | 8.00 | 16.00 |

**Figure S34**. Decay plots for the *Z*-MOAB isomer with respect to order of decay.

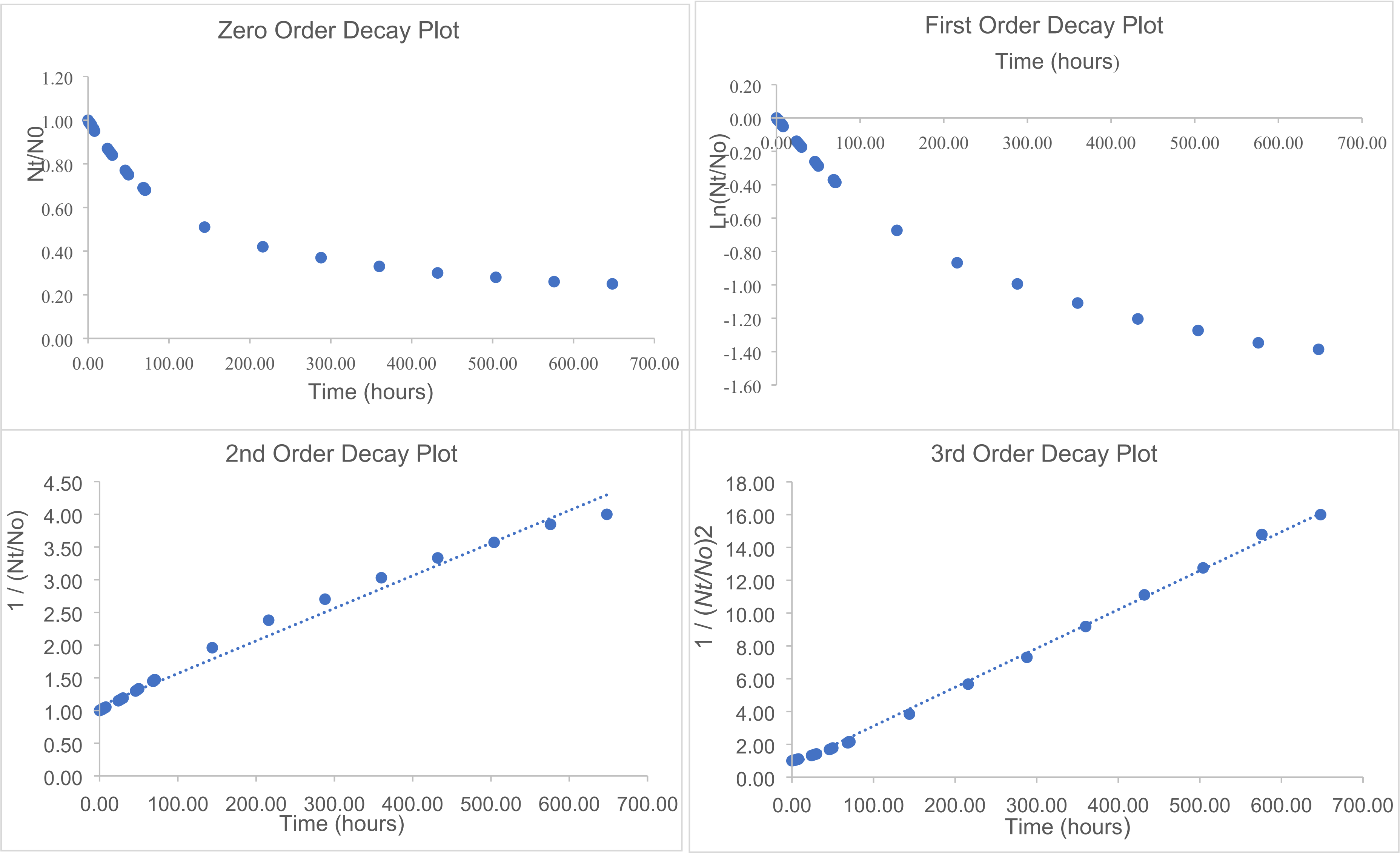

Supplement: SC-013-D2SC00632D-s001 [file SC-013-D2SC00632D-s001.pdf]
